# Conformation-Specific Rate Coefficients of CH_3_CHOO + HCl Determined with Multiple IR/UV Absorption Probes

**DOI:** 10.1021/acs.jpclett.5c01353

**Published:** 2025-06-17

**Authors:** Tang-Yu Kao, Chen-An Chung, Yuan-Pern Lee

**Affiliations:** † Department of Applied Chemistry and Institute of Molecular Science, 34914 National Yang Ming Chiao Tung University, Hsinchu 300093, Taiwan; ‡ Center for Emergent Functional Matter Science, National Yang Ming Chiao Tung University, Hsinchu 300093, Taiwan

## Abstract

Reactions between Criegee intermediates and hydrogen halides significantly impact atmospheric chemistry, particularly in polluted urban environments. We employed a multipass absorption flow cell with four probes to determine the rate coefficient of *syn*- and *anti*-CH_3_CHOO + HCl. Infrared quantum-cascade lasers near 883 and 1280 cm^–1^ provided temporal profiles of *syn*-CH_3_CHOO and a combination of *syn*-CH_3_CHOO and the hydrogen-transferred adduct chloroethyl hydroperoxide (CEHP), respectively. A light-emitting diode at 286 nm probed the precursor CH_3_CHI_2_, hence [CH_3_CHI]_0_ upon photolysis at 248 nm. Laser light at 335 nm probed both *syn*- and *anti*-CH_3_CHOO. By modeling the observed temporal profiles, we derived rate coefficients of *syn*-CH_3_CHOO + HCl and *anti*-CH_3_CHOO + HCl at 298 K as *k*_HCl_^*syn*^ = (5.1 ± 1.1) × 10^–11^ cm^3^ molecule^–1^ s^–1^ and *k*_HCl_^*anti*^ = (2.9 ± 0.8) × 10^–10^ cm^3^ molecule^–1^ s^–1^, respectively. The latter, previously unreported, is ∼ 5.7 times *k*_HCl_^*syn*^, highlighting the conformation-specific reactivity.

## Graphical abstract

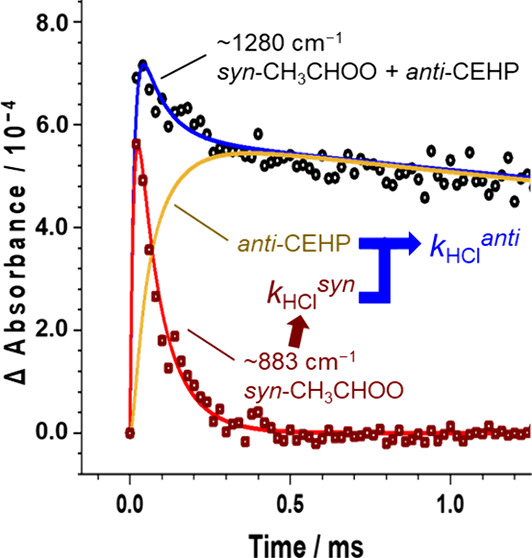

Hydrogen chloride (HCl) is a significant source of chlorine (Cl) atoms in the atmosphere, which play important roles in air quality^1^ through reactions with ozone,^2^ alkanes,^3^ and volatile organic compounds (VOC).^4^ Field measurements indicate that the average mixing ratio of HCl in polluted urban areas ranges from 0.53 to 2.7 ppbv (part per billion by volume), with approximately 45% of Cl atoms in these regions originating from the reaction of HCl with hydroxyl radicals (OH).^5,6^ HCl is directly emitted from industrial processes associated with power generation using chloride-containing fuels, as well as the acid displacement of sea-salt aerosols.^7,8^ The reaction between the Criegee intermediate CH_2_OO and HCl has a large rate coefficient, (4.6–4.8) × 10^–11^ cm^3^ molecule^–1^ s^–1^,^9,10^ which is consistent with theoretical predictions.^11^ The effective first-order rate coefficient of this reaction is comparable to that of CH_2_OO reacting with organic acids (∼2.4 s^–1^),^12^ highlighting the potential importance of CH_2_OO + HCl in atmospheric chemistry. The reported reaction product is a hydrogen-transferred adduct, *gauche*-chloromethyl hydroperoxide (CMHP, CH_2_ClOOH), which has been identified using microwave^13^ and infrared absorption spectroscopy.^10^

In the atmosphere, ozone reacts with 2-alkenes to produce acetaldehyde oxide (CH_3_CHOO), which exists as two conformers: *syn*-CH_3_CHOO and *anti*-CH_3_CHOO. The *syn*-conformer is lower in energy than the *anti*-conformer by 11–16 kJ mol^–1^.^14–16^ Interconversion of these two conformers under ambient conditions is unlikely due to a large barrier of ∼160 kJ mol^–1^.^16,17^ Taatjes et al. distinguished these conformers based on their ionization thresholds, ∼9.4 eV for *syn*-CH_3_CHOO and ∼9.3 eV *anti*-CH_3_CHOO.^18^ Nakajima and Endo further characterized their rotational transitions with microwave spectroscopy.^16,17^ Taking advantage of the significantly greater reactivity of *anti*-CH_3_CHOO toward H_2_O and SO_2_, Sheps et al. determined UV absorption spectra for *syn*-CH_3_CHOO and *anti*-CH_3_CHOO, identifying broad bands peaking near 323 and 360 nm, respectively.^19^ Our group previously reported infrared (IR) spectra of CH_3_CHOO recorded using a step-scan Fourier-transform infrared (FTIR) spectrometer, though severe overlaps prevented distinguishing bands of the *syn*- and *anti*-conformers.^20^ Later, Luo et al. employed a quantum-cascade laser in the region 880–932 cm^–1^ with a resolution of 0.0015 cm^–1^ to record the OO-stretching band of CH_3_CHOO.^21^ After the introduction of CH_3_OH to selectively deplete *anti*-CH_3_CHOO, several spectral lines corresponding exclusively to *syn*-CH_3_CHOO were identified. Liu et al. reported several absorption bands of *syn*-CH_3_CHOO in the near-infrared (NIR) region by detecting OH, an NIR-induced unimolecular decomposition product, using laser-induced fluorescence;^22^ however, no bands of *anti*-CH_3_CHOO could be determined with this method.

CH_3_CHOO has been reported to show conformation-specific reactivities.^23,24^ The unimolecular decomposition of *syn*-CH_3_CHOO is significantly faster than that of *anti*-CH_3_CHOO due to the interaction between the terminal O atom and the H atoms in the methyl moiety of *syn*-CH_3_CHOO.^16,22,25,26^ In contrast, *anti*-CH_3_CHOO is considerably more reactive than *syn*-CH_3_CHOO toward various species, including H_2_O/(H_2_O)_2_,^18,19,27–30^ CH_3_OH,^31–33^ NO_2_,^18,34^ SO_2_,^15,18,19,35–37^ HC­(O)­OH,^12^ CH_3_C­(O)­OH,^12^ amines,^38,39^ and amino alcohol.^39,40^ Kinetic studies of the reactions of *syn*-/*anti*-CH_3_CHOO have typically utilized multiplex photoionization mass spectrometry (MPIMS) or UV absorption techniques. In MPIMS, ionization potentials of 10.5 and 9.37 eV were employed for detecting *syn*- and *anti*-CH_3_CHOO, respectively, with the former accounting for ∼80% of *syn*-CH_3_CHOO.^18^ UV absorption below 360 nm was commonly used to monitor both *syn*- and *anti*-CH_3_CHOO.^19^ Rate coefficients were derived either by observing the rapid decay attributed to *anti*-CH_3_CHOO^41^ or by fitting the decay profile with a rapid and a slow components corresponding to *anti*- and *syn*-CH_3_CHOO, respectively.^19^ Lade et al. used reference spectra of CH_3_CHI_2_, *syn*- and *anti*-CH_3_CHOO, and IO to fit observed UV spectra at each time interval to determine the concentration of each species during the reaction and obtained the temporal profiles of *syn*- and *anti*-CH_3_CHOO; this deconvolution enabled them to derive the respective rate coefficients.^36^ Recently, Kao et al. in our group developed an IR/UV-dual-probe system to investigate the reactions of *syn*- and *anti*-CH_3_CHOO.^42^ By comparing temporal profiles obtained through IR absorption specific to *syn*-CH_3_CHOO and UV absorption covering both *syn*- and *anti*-CH_3_CHOO, these authors determined the temporal profile of *anti*-CH_3_CHOO. With this approach, the rate coefficient for the self-reaction of *anti*-CH_3_CHOO was determined to be (6 ± 2) × 10^–10^ cm^3^ molecule^–1^ s^–1^, ∼4 times the value for the self-reaction of *syn*-CH_3_CHOO, (1.4 ± 0.3) × 10^–10^ cm^3^ molecule^–1^ s^–1^. Additionally, the rate coefficient for the cross-reaction between *syn*-CH_3_CHOO and *anti*-CH_3_CHOO was estimated to be (2.1 ± 0.6) × 10^–10^ cm^3^ molecule^–1^ s^–1^.

In the reaction of CH_3_CHOO with HCl in a discharged jet, Cabezas and Endo identified both *syn*- and *anti*-conformers of the product, chloroethyl hydroperoxide (CEHP, CH_3_CHClOOH), using microwave spectroscopy.^43^ In the reaction of CH_3_CHOO with HCl at ambient temperature, Su and Lee in our group assigned ten transient absorption bands of *anti*-CEHP using a step-scan FTIR spectrometer.^44^ By introducing methanol to react more rapidly with *anti*-CH_3_CHOO and thereby altering the ratio of *syn*-CH_3_CHOO to *anti*-CH_3_CHOO in the system, these authors observed the IR spectrum exclusively of *anti*-CEHP with a reduced proportion, suggesting that *syn*-CEHP, produced from *syn*-CH_3_CHOO + HCl, readily converts to *anti*-CEHP under their experimental conditions at 298 K.

Liu et al. employed a flow tube to investigate the reaction of CH_3_CHOO with HCl at 10 Torr and reported the rate coefficient for *syn*-CH_3_CHOO + HCl as *k*_HCl_^*syn*^ = (4.77 ± 0.95) × 10^–11^ cm^3^ molecule^–1^ s^–1^ at 298 K by probing OH with laser-induced fluorescence; OH was produced solely from the unimolecular decomposition of *syn*-CH_3_CHOO.^45^ Cabezas and Endo further noted that the reaction of *anti*-CH_3_CHOO + HCl was significantly faster than that of *syn*-CH_3_CHOO + HCl; despite an initial concentration ratio of *anti*-CH_3_CHOO to *syn*-CH_3_CHOO of ∼ 1:5 under their experimental conditions, the estimated ratio of products *anti*-CEHP to *syn*-CEHP was ∼3:1.^43^ Su and Lee observed the formation of *anti*-CEHP and estimated an overall rate coefficient for the reaction of *syn*-/*anti*-CH_3_CHOO with HCl from FTIR experiments as *k*_HCl_^*syn*/*anti*^ = (2.7 ± 1.0) × 10^–10^ cm^3^ molecule^–1^ s^–1^ at 298 K,^44^ six times that reported for *syn*-CH_3_CHOO + HCl by Liu et al.,^45^ indicating that *anti*-CH_3_CHOO reacts with HCl much faster than *syn*-CH_3_CHOO. However, no direct measurement of the rate coefficient for *anti*-CH_3_CHOO + HCl has been reported.

In this study, we extended the IR/UV-dual-probe experimental setup^42^ further by incorporating a second QCL near 1280 cm^–1^ to perform kinetic measurements for the reaction of CH_3_CHOO with HCl. The probe at 286 nm monitored [CH_3_CHI_2_] upon photolysis, determining the initial [CH_3_CHI]_0_. The probe at 335 nm measured the absolute concentrations and tracked the temporal evolution of *syn*-/*anti*-CH_3_CHOO. The QCL near 883 cm^–1^ captured the temporal evolution of *syn*-CH_3_CHOO, while the QCL near 1280 cm^–1^ probed the temporal profile of both *syn*-CH_3_CHOO and the reaction product *anti*-CEHP. By employing various methods and fitting experimental data to a chemical model, we determined the rate coefficients for *syn*-CH_3_CHOO + HCl and *anti*-CH_3_CHOO + HCl.

Following the report of Luo et al., we probed spectral lines attributed exclusively to *syn*-CH_3_CHOO near 883 cm^–1^.^21^ The absorbance temporal profiles of *syn*-CH_3_CHOO were monitored in spectral regions 883.105–883.135 and 883.148–883.185 cm^–1^. The initial concentrations of *syn*-CH_3_CHOO and *anti*-CH_3_CHOO, denoted [*syn*-CH_3_CHOO]_0_ and [*anti*-CH_3_CHOO]_0_, were estimated based on [CH_3_CHI]_0_, determined from the 286 nm probe, and the reported branching ratios of 0.86 × 0.80 and 0.86 × 0.20, respectively. The branching ratio of 0.86 for the production of *syn*- and *anti*-CH_3_CHOO is taken from Howes et al.,^35^ and the branching ratio of 0.80:0.20 for *syn*-CH_3_CHOO: *anti*-CH_3_CHOO is taken from Kao et al.^42^ Temporal profiles derived from the IR probe near 883 cm^–1^ were subsequently converted to concentrations for the kinetic analysis of *syn*-CH_3_CHOO.

The experimental conditions of 17 experiments conducted in six sets are summarized in Table S1, Supporting Information. Representative temporal profiles of concentrations of *syn*-CH_3_CHOO in experimental set 3 at various initial concentrations of HCl (ranging from 6.6 × 10^13^ to 18.6 × 10^13^ molecules cm^–3^) and a total pressure of 8.0 Torr are shown in Figure S1. The initial concentration of *syn*-/*anti*-CH_3_CHOO was estimated to be 7.2 × 10^12^ molecules cm^–3^.

We employed the previously reported kinetic model,^42^ which includes three channels of the formation reaction, self-reactions of *syn*- and *anti*-CH_3_CHOO, the cross-reaction between *syn*-CH_3_CHOO and *anti*-CH_3_CHOO, and possible secondary reactions involving CH_3_CHOO and CH_3_CHIOO. Additionally, the title reactions *syn*-CH_3_CHOO + HCl and *anti*-CH_3_CHOO + HCl were incorporated into the mechanism, as detailed in Table S2. More detailed discussions on the fitting can be found in Supporting Information, Note SA.

According to the report by Su and Lee,^44^ the reaction products for both *syn*- and *anti*-CH_3_CHOO with HCl are *anti*-CEHP,

1a $$syn‐{\mathrm{CH}}_{3}\mathrm{CHOO}+\mathrm{HCl}\to anti‐\mathrm{CEHP}$$

1b $$anti‐{\mathrm{CH}}_{3}\mathrm{CHOO}+\mathrm{HCl}\to anti‐\mathrm{CEHP}$$

The pseudo-first-order rate coefficient for reaction 1a, *k*_*syn*_^I^ = *k*_HCl_^*syn*^ × [HCl]_0_, was determined through fitting. Since we probed [*syn*-CH_3_CHOO], the rate coefficient of reaction 1b (*k*_HCl_^*anti*^) had an insignificant impact on the fitting. However, for completeness, *k*_HCl_^*anti*^ was set to 2.9 × 10^–10^ cm^3^ molecule^–1^ s^–1^, to be discussed later.

A summary of the fitted first-order rate coefficients (*k*_*syn*_^I^) from experiments conducted under the following conditions is presented in Table S1: [CH_3_CHI_2_]_0_ = (3.7–17.2) × 10^14^ molecules cm^–3^, [CH_3_CHI]_0_ = (8.1–55.1) × 10^12^ molecules cm^–3^, [HCl]_0_ = (6.6–55.0) × 10^13^ molecules cm^–3^, total pressure *P*_T_ = 4–16 Torr, and *T* = 298 K. Figure 1 illustrated the dependence of the fitted *k*_*syn*_^I^ on [HCl]_0_. From the slope of this plot, the bimolecular rate coefficient of *syn*-CH_3_CHOO + HCl was determined to be *k*_HCl_^*syn*^ = (5.06 ± 0.10) × 10^–11^ cm^3^ molecule^–1^ s^–1^; the listed error represents one standard deviation in fitting. When the fitted line was constrained to pass through the origin, *k*_HCl_^*syn*^ was calculated to be (5.50 ± 0.10) × 10^–11^ cm^3^ molecule^–1^ s^–1^. After considering possible errors, as discussed in Note SA (Supporting Information), we estimated the overall standard error to be ∼21%. The bimolecular rate coefficient for *syn*-CH_3_CHOO + HCl is thus reported as *k*_HCl_^*syn*^ = (5.1 ± 1.1) × 10^–11^ cm^3^ molecule^–1^ s^–1^. This value aligns with the only available literature value, (4.77 ± 0.95) × 10^–11^ cm^3^ molecule^–1^ s^–1^, reported by Liu et al.,^45^ who detected OH produced from the unimolecular decomposition of *syn*-CH_3_CHOO using laser-induced fluorescence. While Liu et al. studied a narrow range of [HCl], (0.2–1.4) × 10^13^ molecules cm^–3^, our experiments covered a significantly broader range, with [HCl] = (6.6–55.0) × 10^13^ molecules cm^–3^.

**Figure 1:**
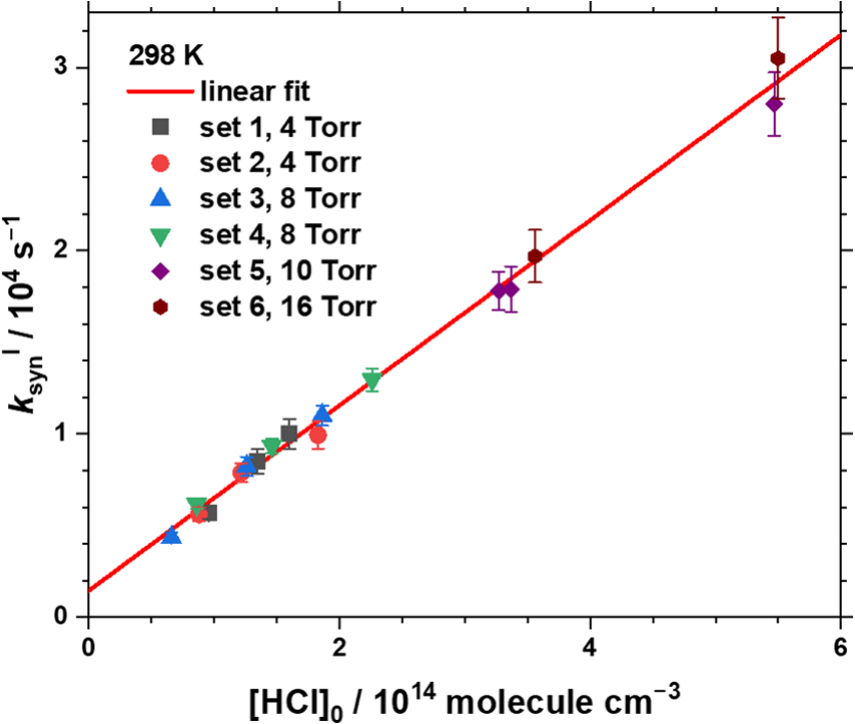
Pseudo-first-order rate coefficient *k*_*syn*_^I^ as a function of [HCl]_0_. Total pressure 4–16 Torr, *T* = 298 K, [CH_3_CHI]_0_ = (8.1–55.1) × 10^12^ molecules cm^–3^, and [HCl]_0_ = (6.6–55.0) × 10^13^ molecules cm^–3^. The linear fit yields the red line with a slope of *k*_HCl_^*syn*^ = (5.06 ± 0.10) × 10^–11^ cm^3^ molecule^–1^ s^–1^ and an intercept of 1430 ± 260 s^–1^. The error bars indicate only the uncertainties in fitting *k*_*syn*_^I^.

For the reaction of *anti*-CH_3_CHOO with HCl, we utilized a second QCL near 1280 cm^–1^ to detect *syn*-CH_3_CHOO and the product *anti*-CEHP (CH_3_CHClOOH), as detailed in Supporting Information Note SB, including Figures S2–S6. As the molecule becomes larger, spectral assignments become extremely challenging, especially when the overtone (2ν_11_) and combination bands (such as ν_9_ + ν_12_) significantly perturbed the ν_7_ band of CH_3_CHOO in this region. Although exact spectral assignments could not be achieved, we did locate several lines in regions 1279.93–1279.95, 1279.87–1279.89, and 1279.80–1279.82 cm^–1^ that have the same temporal behavior as those of *syn*-CH_3_CHOO near 883 cm^–1^. These features were used to probe *syn*-CH_3_CHOO near 1280 cm^–1^.

The IR spectrum of *anti*-CEHP, as reported by Su and Lee, featured a B_4_ band in the region 1250–1290 cm^–1^ with a Q-band at 1271.8 cm^–1^,^44^ overlapping with the ν_7_ band of CH_3_CHOO near 1280 cm^–1^. Temporal profiles of a combination of *syn*-CH_3_CHOO and *anti*-CEHP were thus measured using the QCL near 1280 cm^–1^, and their kinetic behavior was fitted to a kinetic model. The overall strategy is to use the measured [*syn*-CH_3_CHOO]_0_ and the determined rate coefficient of *k*_HCl_^*syn*^ to simulate the temporal profile of *syn*-CH_3_CHOO, so that it can be combined with the simulated temporal profile of *anti*-CEHP to fit with the experimental observation (Method A). Furthermore, we employed two additional methods to validate this approach. Method B measured the temporal profile of *syn*-CH_3_CHOO near 883 cm^–1^ instead of simulating it and converted it to that of *syn*-CH_3_CHOO near 1280 cm^–1^, whereas Method C derived the temporal profile of *anti*-CH_3_CHOO by comparison of UV profiles at 335 nm (monitoring both *syn*- and *anti*-CH_3_CHOO) with IR profiles ∼883 cm^–1^ (monitoring only *syn*-CH_3_CHOO).

Since both *syn*-CH_3_CHOO and *anti*-CEHP absorb at this wavenumber, but with different cross sections, one needs to incorporate the ratios of integrated absorbance σ_*syn*_^1280^/σ_CEHP_^1280^ to convert simulated concentrations of *syn*-CH_3_CHOO and *anti*-CEHP to absorbance for proper fitting. As discussed in Supporting Information Note SC (Figure S7 and S8 and Tables S3 and S4), σ_*syn*_^1280^/σ_CEHP_^1280^ was determined to be 1.06 ± 0.11.

When we applied the model in Table 1 but assumed *k*_HCl_^*syn*^ = *k*_HCl_^*anti*^ to fit *k*_HCl_^*syn*^ (=*k*_HCl_^*anti*^), the observed temporal profiles could not be fitted properly, as shown in representative fittings in Figure S9. The fitted values of *k*_HCl_^*syn*^ (=*k*_HCl_^*anti*^) were generally larger than the literature value^45^ or the value determined in this work, *k*_HCl_^*syn*^ = (4.8–5.1) × 10^–11^ cm^3^ molecule^–1^ s^–1^. Additionally, the decay could not be accounted for properly. These results suggest that *k*_HCl_^*syn*^ and *k*_HCl_^*anti*^ values are significantly different.

**Table 1:** Kinetic Model for Fitting the Reaction of *anti*-CH_3_CHOO + HCl

reaction	rate coefficient^a^,^b^		reference
CH_3_CHI + O_2_ → *syn*-CH_3_CHOO + I	*k*_form_^a^	*x* × *y* × 3.8 × 10^–12^ (±18%)	^19^,^42^
		*x* = 0.86; *y* = 0.80	
CH_3_CHI + O_2_ → *anti*-CH_3_CHOO + I	*k*_form_^b^	*x* × (1 – *y*) × 3.8 × 10^–12^ (±18%)	^19^,^42^
CH_3_CHI + O_2_ → CH_3_CHIOO	*k*_form_^c^	(1 – *x*) × 3.8 × 10^–12^ (±18%)	^35^
2*syn*-CH_3_CHOO → 2CH_3_CHO + O_2_	*k*_self_^*syn*^	1.4 × 10^–10^ (±20%)	^42^
2*anti*-CH_3_CHOO → 2CH_3_CHO + O_2_	*k*_self_^*anti*^	6 × 10^–10^ (±30%)	^42^
*syn*-CH_3_CHOO + *anti*-CH_3_CHOO → 2CH_3_CHO + O_2_	*k*_self_^*cross*^	2.1 × 10^–10^ (±30%)	^42^
*syn*-CH_3_CHOO + HCl → *anti*-CEHP	*k*_HCl_^*syn*^	5.1 × 10^–11^ (±20%)	This work
*anti*-CH_3_CHOO + HCl → *anti*-CEHP	*k*_HCl_^*anti*^	fitted	
*anti*-CEHP → loss	*k*_loss_	fitted	
*syn*-CH_3_CHOO + I → products	*k*_1_	9.0 × 10^–12^	^46^
*anti*-CH_3_CHOO + I → products	*k*_2_	9.0 × 10^–12^	^46^
CH_3_CHIOO + I → CH_3_CHIO + IO	*k*_3_	3.5 × 10^–11^	^46^
2CH_3_CHIOO → 2ICH_3_CHO + O_2_	*k*_4_	9.0 × 10^–11^	^46^
CH_3_CHIO → CH_3_CHO + I	*k*_*5*_	10^6^ s^–1^	^47^
2IO → I_2_ + O_2_	*k*_6_	9.9 × 10^–11^	^46^

a Listed second-order rate coefficients are in cm^3^ molecule^–1^ s^–1^, unless noted.

b *x* is the branching ratio of *syn*-CH_3_CHOO and *anti*-CH_3_CHOO in total products and *y* is the branching ratio of *syn*-CH_3_CHOO in total CH_3_CHOO.

The temporal profiles observed near 1280 cm^–1^ (for both *syn*-CH_3_CHOO and *anti*-CEHP) were fitted by using the model listed in Table 1 (Method A). This model is identical to that in Table S2, except that *k*_HCl_^*syn*^ was set at (5.1 ± 1.1) × 10^–11^ cm^3^ molecule^–1^ s^–1^ while *k*_HCl_^*anti*^ was fitted, and the loss of *anti*-CEHP was included in the model as *anti*-CEHP was also monitored. For convenience, instead of using the σ_*syn*_^1280^/σ_CEHP_^1280^ ratio determined in this work, we employed two free-fitting conversion factors *f*_CI_ and *f*_CEHP_ to convert concentrations of *syn*-CH_3_CHOO and *anti*-CEHP, respectively, to absorbance and compare *f*_CI_/*f*_CEHP_ with σ_*syn*_^1280^/σ_CEHP_^1280^ (Table S5).

The temporal profiles of bands near 1280 cm^–1^ recorded in experimental set 2 are presented in Figure 2. The experimental conditions include an initial concentration of [CH_3_CHI_2_]_0_ = 21–29 mTorr, [HCl]_0_ ranging from 1.2 to 17.1 mTorr, equivalent to (0.4–5.5) × 10^14^ molecules cm^–3^, a total pressure of 10 Torr, and a temperature of 298 K. Symbols represent experimental data, while lines depicted simulated absorbance profiles for *syn*-CH_3_CHOO + *anti*-CEHP. Figure 3 illustrates representative simulated temporal profiles of the total absorbance of *syn*-CH_3_CHOO and *anti*-CEHP (blue), along with the simulated absorbance profiles of *syn*-CH_3_CHOO (red) and *anti*-CEHP (gold) and the experimental data (black circles). The relative maximum of *anti*-CEHP compared to *syn*-CH_3_CHOO increases as [HCl]_0_ increases, indicating that *syn*-CH_3_CHOO is rapidly consumed, leading to *anti*-CEHP becoming the dominant absorbance at large amounts of [HCl]_0_. At [HCl]_0_ above 10 mTorr (Figure 3d), the experimental decay of *syn*-CH_3_CHOO is nearly offset by the rising component of *anti*-CEHP, rendering the decay component indistinguishable.

**Figure 2:**
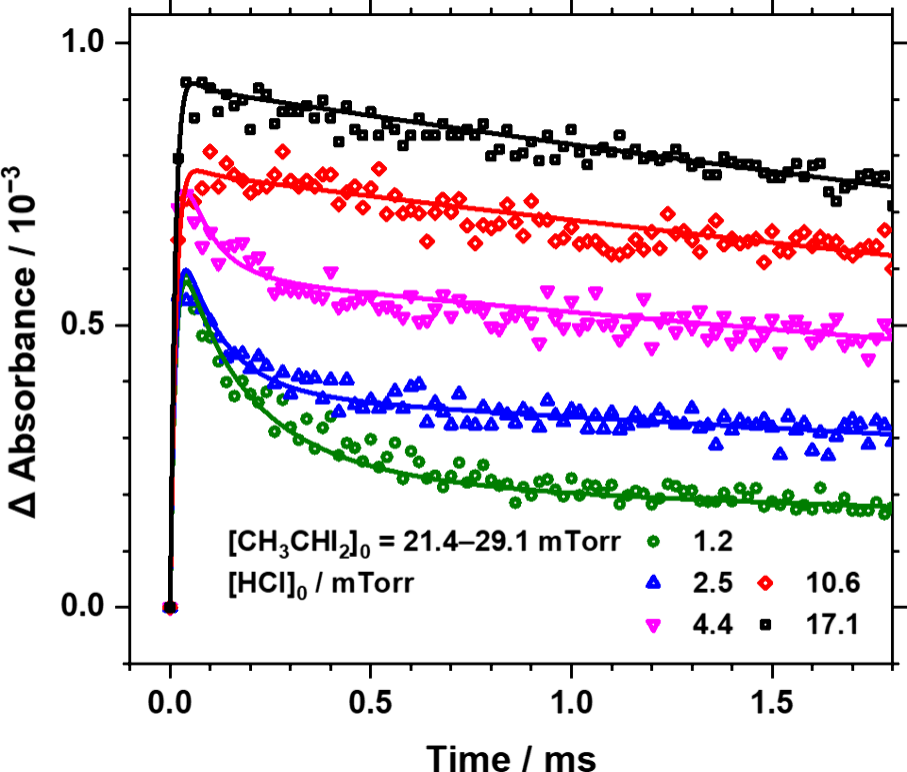
Comparison of observed temporal profiles of *syn*-CH_3_CHOO + *anti*-CEHP in experimental set 2 with simulated profiles (solid lines) of *syn*-CH_3_CHOO + *anti*-CEHP based on Method A. Total pressure *P*_T_ = 10 Torr and *T* = 298 K. Partial pressures of CH_3_CHI_2_ are 21.4–29.1 mTorr and those of HCl are 1.2–17.1 mTorr. Integrated absorbance in regions 1279.93–1279.95, 1279.87–1279.89, and 1279.80–1279.82 cm^–1^ was probed.

**Figure 3:**
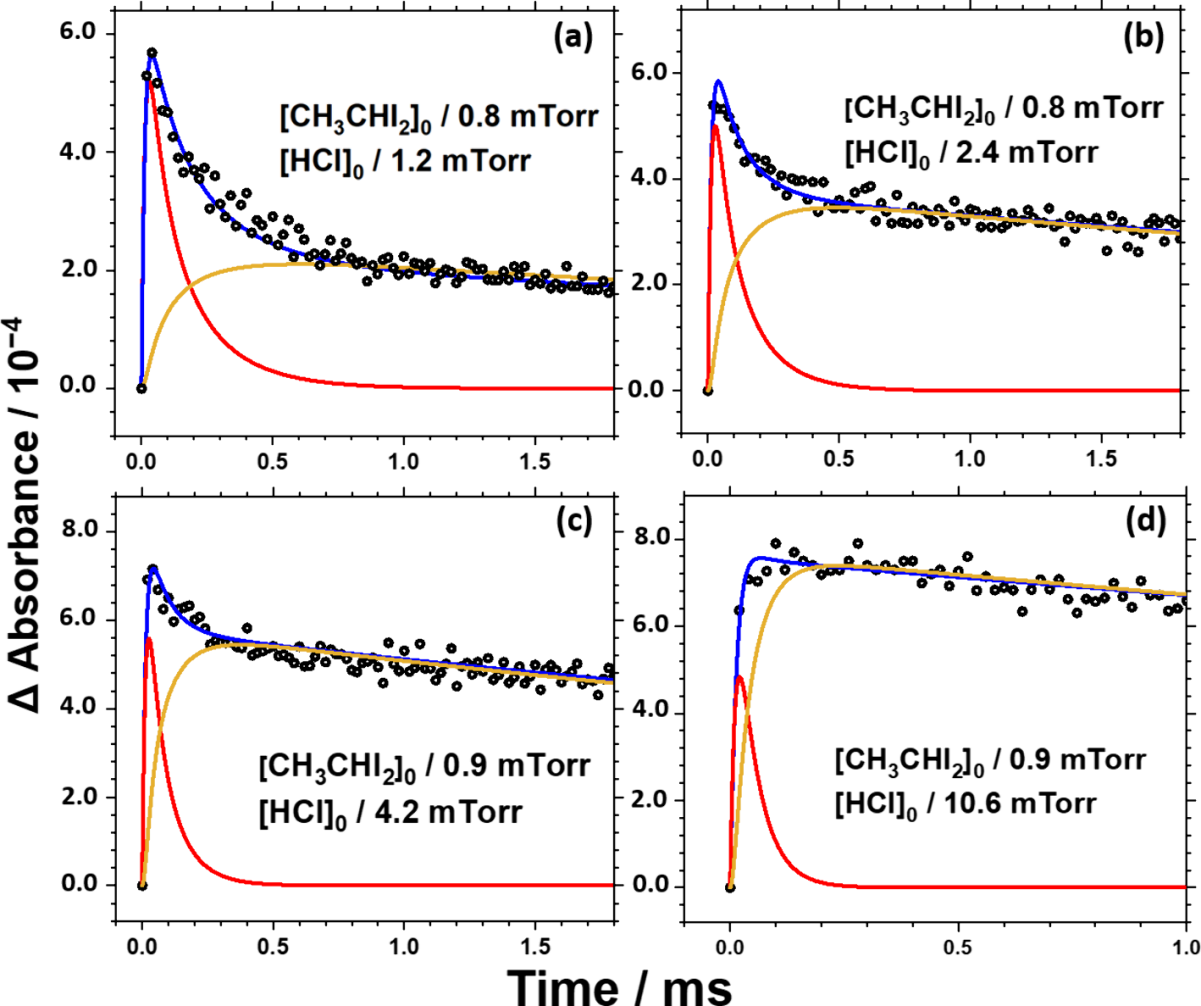
Individual simulated absorbance temporal profiles of *syn*-CH_3_CHOO (red line) and *anti*-CEHP (gold line). The experimental absorbance is shown in black circles, and the simulated total absorbance of *syn*-CH_3_CHOO + *anti*-CEHP is presented with blue lines. Total pressure *P*_T_ = 10 Torr and *T* = 298 K. [HCl]_0_ = 1.2 (a), 2.4 (b), 4.2 (c), and 10.6 mTorr (d).

A summary of the experimental conditions and the fitted results from 16 measurements at 298 K in three sets is presented in Table S5. The experiments were conducted with [CH_3_CHI_2_]_0_ = (6.9–17.2) × 10^14^ molecules cm^–3^, [CH_3_CHI]_0_ = (2.4–5.5) × 10^13^ molecules cm^–3^, [HCl]_0_ = (0.4–9.1) × 10^14^ molecules cm^–3^ (1.2 to 28.2 mTorr), and total pressure between 10 to 16 Torr. The fitted ratio of *f*_CI_/*f*_CEHP_ are also listed in Table S5, with an average value of 0.99 ± 0.10, which is in agreement with the independently derived value σ_*syn*_^1280^/σ_CEHP_^1280^ = 1.06 ± 0.11 described above. This consistency validates the robustness of the fitting process.

The fitted first-order rate coefficients (*k*_*anti*_^I^) versus [HCl]_0_ are depicted in Figure 4, from which the bimolecular rate coefficient (*k*_HCl_^*anti*^) was obtained. Linear fitting of Figure 4 yields *k*_HCl_^*anti*^ = (2.93 ± 0.13) × 10^–10^ cm^3^ molecule^–1^ s^–1^, which adjust to *k*_HCl_^*anti*^ = (2.96 ± 0.10) × 10^–10^ cm^3^ molecule^–1^ s^–1^ when the line is constrained to pass through the origin. The loss rate coefficients of CEHP, *k*_loss_, were fitted to be 127 ± 6 s^–1^ (Table S5), attributable partly to the pumping loss and partly to the unimolecular decomposition of CEHP, as was also reported by Su and Lee.^44^ The small loss rate of CEHP does not affect the fitted first-order rate coefficient of *k*_*anti*_^I^.

**Figure 4:**
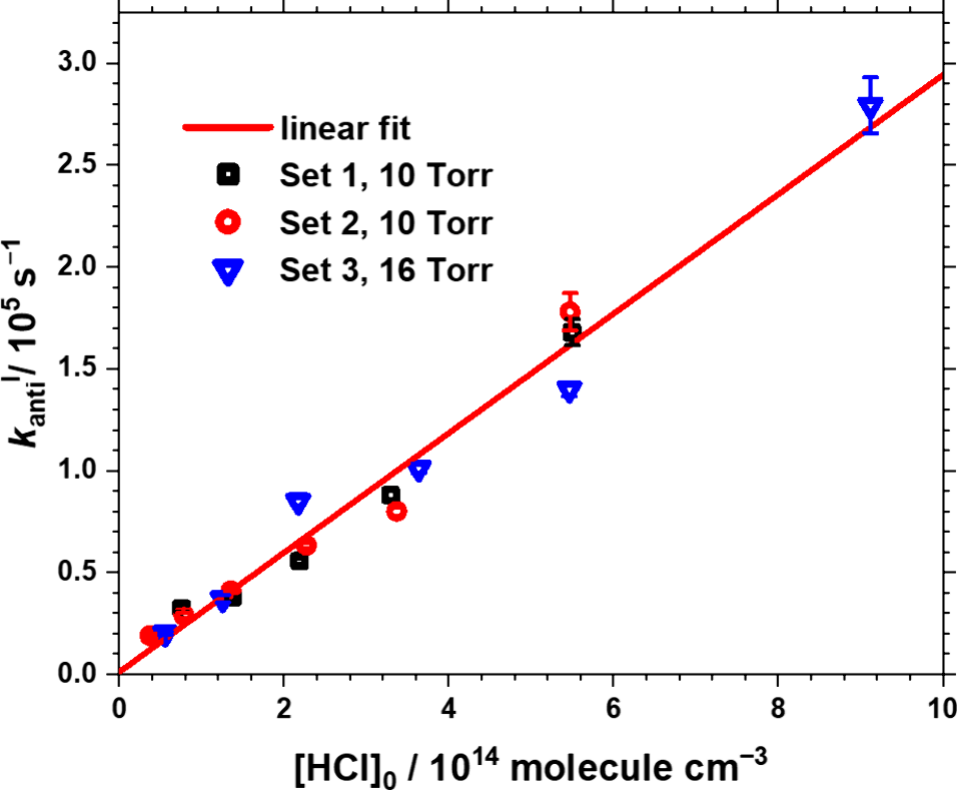
First-order rate coefficient *k*_*anti*_^I^ as a function of the concentration of [HCl]_0_. Total pressure *P*_T_ = 10–16 Torr, *T* = 298 K, [CH_3_CHI]_0_ = (2.4–5.5) × 10^13^ molecules cm^–3^, [HCl]_0_ = (0.4–9.1) × 10^14^ molecules cm^–3^. The linear fit yields the red line with a slope of *k*_HCl_^*anti*^ = (2.93 ± 0.13) × 10^–10^ cm^3^ molecule^–1^ s^–1^ and an intercept of (1100 ± 4610) s^–1^. The error bars indicate only the uncertainties in fitting the *k*_*anti*_^I^.

Considering the errors associated with the estimation of concentrations of *syn*-/*anti*-CH_3_CHOO (±20%) that translates to an error of ±13% in *k*_HCl_^*anti*^, the error in *k*_form_ of the formation reaction (±18%) that translates to an error of ±4% in *k*_HCl_^*anti*^, the error in *k*_self_^*syn*^ of the self-reaction of *syn*-CH_3_CHOO (±20%) that translates to an error of ±8% in *k*_HCl_^*anti*^, the error in *k*_self_^*cross*^ of the cross reaction of *syn*- and *anti*-CH_3_CHOO (±30%) that translates to an error of ±3% in *k*_HCl_^*anti*^, the error in *k*_self_^*anti*^ of the self-reaction of *anti*-CH_3_CHOO (±30%) that translates to an error of ±15% in *k*_HCl_^*anti*^, the error in *k*_HCl_^*syn*^ for the reaction of *syn*-CH_3_CHOO + HCl (±20%) that translates to an error of ±14% in *k*_HCl_^*anti*^, the fitting error of ±5%, and other typical errors in measurements of flow rates (3%), temperature (1%), and pressure (3%), we estimated the overall standard error to be ±27%. Consequently, the bimolecular rate coefficient for *anti*-CH_3_CHOO + HCl is reported as *k*_HCl_^*anti*^ = (2.9 ± 0.8) × 10^–10^ cm^3^ molecule^–1^ s^–1^ at 298 K.

We employed two additional methods to confirm our results. In Method B, discussed in Supporting Information Note SD, the temporal profiles of *anti*-CEHP near 1280 cm^–1^ were obtained by subtracting the temporal profiles of *syn*-CH_3_CHOO near 1280 cm^–1^ (recorded with the QCL near 883 cm^–1^ and converted by the cross-section ratio σ_*syn*_^1280^/σ_*syn*_^883^) from those of *syn*-CH_3_CHOO + *anti*-CEHP determined with the QCL near 1280 cm^–1^. The rise of *anti*-CEHP was subsequently fitted with the kinetic model. The *k*_*anti*_^I^ values derived for experimental set 2 using this method are listed in Table S5 for comparison with those obtained using Method A. The average absolute deviation was 9.5 ± 12.2%, reflecting possible fitting errors between these two methods. Method C compared UV profiles at 335 nm (monitoring both *syn*- and *anti*-CH_3_CHOO) with IR profiles of ∼883 cm^–1^ (monitoring only *syn*-CH_3_CHOO) to derive the temporal profiles of *anti*-CH_3_CHOO, which were then fitted using the kinetic model, as detailed in Supporting Information Note SE. Results from this method were also compared with those from Method A in Table S5. The average absolute deviation from Method A was 21 ± 17%, attributed to the larger errors of the small component of *anti*-CH_3_CHOO derived by subtracting two large values. Nonetheless, the agreement remained within the overall uncertainties (±27%) discussed above, supporting the presence of *anti*-CH_3_CHOO in the system and its greater reactivity toward HCl.

Su and Lee, from our group, utilized a step-scan FTIR spectrometer to measure the overall formation rate of *anti*-CEHP from *syn*-/*anti*-CH_3_CHOO + HCl. They reported an effective rate coefficient of (2.7 ± 1.0) × 10^–10^ cm^3^ molecule^–1^ s^–1^, based on the fitting to a simple exponential rise.^44^ In that study, these authors assumed that the effective rate coefficient for *syn*-/*anti*-CH_3_CHOO (*k*_HCl_^*syn/anti*^) could be expressed as *k*_HCl_^*syn/anti*^ = *a* × *k*_HCl_^*syn*^ + *b* × *k*_HCl_^*anti*^, in which *a* and *b* are the branching ratios of *syn*- and *anti*-CH_3_CHOO. Using this approach, these authors estimated *k*_HCl_^*anti*^ ≈ 8 × 10^–10^ cm^3^ molecule^–1^ s^–1^ from *k*_HCl_^*syn*^ = 4.8 × 10^–11^ cm^3^ molecule^–1^ s^–1^,^45^ *k*_HCl_^*syn/anti*^ = 2.7 × 10^–10^ cm^3^ molecule^–1^ s^–1^, and *a*:*b* = 0.7:0.3.^19^ Actually, this approach is inappropriate as the apparent rate coefficient for the reaction of a mixture of *syn*-/*anti*-CH_3_CHOO with HCl, derived from the rise of the product, cannot be correctly accounted for by *a* × *k*_HCl_^*syn*^ + *b* × *k*_HCl_^*anti*^; the *k*_HCl_^*anti*^ previously derived from *k*_HCl_^*syn/anti*^ is hence incorrect. In any case, the value of *k*_HCl_^*syn/anti*^ should lie between those of *k*_HCl_^*syn*^ and *k*_HCl_^*anti*^. Despite of its large uncertainty, the previously reported experimental value of *k*_HCl_^*syn/anti*^ = (2.7 ± 1.0) × 10^–10^ cm^3^ molecule^–1^ s^–1^ indeed lies between our observation of *k*_HCl_^*syn*^ = (5.1 ± 1.1) × 10^–11^ cm^3^ molecule^–1^ s^–1^ and *k*_HCl_^*anti*^ = (2.9 ± 0.8) × 10^–10^ cm^3^ molecule^–1^ s^–1^ at 298 K.

The measured value of *k*_HCl_^*anti*^ = (2.9 ± 0.8) × 10^–10^ cm^3^ molecule^–1^ s^–1^ at 298 K is approximately 5.7 times *k*_HCl_^*syn*^ = (5.1 ± 1.1) × 10^–11^ cm^3^ molecule^–1^ s^–1^ determined in this work, clearly illustrating the conformation-specific kinetics of CH_3_CHOO + HCl. According to the quantum-chemically computed reaction pathway scheme,^29,44^ the reaction of *syn*-CH_3_CHOO + HCl proceeds via a prereaction complex and a submerged transition structure (energy about −30 kJ mol^–1^ below *syn*-CH_3_CHOO + HCl), forming *syn*-CEHP with exothermicity ∼158 ± 5 kJ mol^–1^. In contrast, the reaction of *anti*-CH_3_CHOO + HCl to produce *anti*-CEHP is barrierless with an exothermicity of ∼177 ± 6 kJ mol^–1^. This difference explains why *k*_HCl_^*anti*^ is significantly greater than *k*_HCl_^*syn*^.

Field observations indicate that the average mixing ratio of gaseous HCl in polluted air is approximately 2 ppbv.^5,6^ The pseudo-first-order decay of *syn*-CH_3_CHOO by HCl is ∼3 s^–1^, which is significantly smaller than the unimolecular decomposition rate coefficient of *syn*-CH_3_CHOO (∼150 s^–1^).^48^ For *anti*-CH_3_CHOO, the pseudo-first-order decay rate coefficient by HCl is ∼14 s^–1^. However, the reactions of *anti*-CH_3_CHOO with H_2_O and (H_2_O)_2_ are remarkably faster, with pseudo-first-order decay rate coefficients of ∼4000 and 8600 s^–1^, respectively.^27,28^ These findings suggest that the reactions of *syn*- and *anti*-CH_3_CHOO with HCl play minor roles in their atmospheric decay. Nevertheless, this study serves as a prototype for understanding fundamental aspects of conformer-specific kinetics of larger Criegee intermediates.

## Methods

The detailed experimental set up has been described elsewhere.^42^ The flow reactor is equipped with a pair of Herriott mirrors (radius of curvature 40 cm, diameter 51 mm) placed 75.4 cm apart. The central part (∼2.5 cm in diameter) of the mirrors was removed to allow the photolysis beam to pass through. Both *syn*- and *anti*-CH_3_CHOO were generated by irradiating a flowing mixture of CH_3_CHI_2_ and O_2_ at 4–16 Torr and 298 K with light at 248 nm from a KrF excimer laser (Coherent, Compex Pro 102F). Typically, the photolysis laser was operated at 7 Hz; the beam size was ∼23 × 15 mm^2^ and energy was ∼140 mJ pulse^–1^ at the reactor entrance.

Beams from two continuous wave (*cw*) external-cavity mode-hop-free quantum-cascade lasers (QCL) with wavelengths ∼8 μm (Daylight Solutions, 41078-MHF, resolution ∼0.002 cm^–1^) and ∼11 μm (Daylight Solutions, 41112-MHF, resolution ∼0.002 cm^–1^) were injected into the Herriott absorption cell to record time-resolved IR absorption spectra in regions of 1263–1335 and 880–932 cm^–1^, respectively. Each measurement employs a scanning step size of approximately 0.002 cm^–1^ and a probing duration of 2.1 s following each wavelength tuning. During this probing duration, the photolysis UV laser was triggered 15 times at 7 Hz, and the transient signal was averaged over 15 measurements at each IR wavelength. The data sampling rate was typically 2–5 M samples s^–1^. For kinetic measurements, instead of fixing the QCL at a specific wavenumber to monitored the absorption, the spectrum near 883 cm^–1^ was scanned, and the *syn*-CH_3_CHOO bands were integrated over the spectral regions 883.105–883.135 and 883.148–883.185 cm^–1^ to mitigate potential wavelength shifts. Similarly, the spectrum was scanned near 1280 cm^–1^ and bands were integrated over spectral regions 1279.93–1279.95, 1279.87–1279.89, and 1279.80–1279.82 cm^–1^.

Two additional UV probes were utilized in the experiments. A light-emitting diode (LED) at 286 ± 7 nm was employed to determine [CH_3_CHI_2_], which was used to calculate the initial concentration of CH_3_CHI, [CH_3_CHI]_0_, upon photolysis at 248 nm, based on the absorption cross-section (3.6 × 10^–18^ cm^2^) of CH_3_CHI_2_ at 286 nm.^49^ A solid-state continuous-wave (*cw*) laser at 335 nm (10 mW) was used to obtain the temporal profiles and absolute concentration of *syn*- and *anti*-CH_3_CHOO; the absorption cross sections reported by Sheps et al. for *syn*- and *anti*-CH_3_CHOO at 335 nm, 11.3 × 10^–18^ and 8.5 × 10^–18^ cm^2^ molecule^–1^, respectively, were used.^19^

Vapor from liquid CH_3_CHI_2_ was carried by O_2_ into an absorption cell (51 cm) located upstream of the reactor. The partial pressure of CH_3_CHI_2_, and hence its fraction in the total flow, was measured using LED light at 286 nm and a Si-photodiode detector. Partial pressures of O_2_, He, and HCl in the reactor were determined from the flow rate of each gas steam and the total pressure. CH_3_CHI_2_ (96–98%, Orgchem Tech.), O_2_ (99.99%, Chiah-Lung), He (99.9995%, Chiah-Lung), and HCl (99.999%, Ching-Fong) were used without further purification.

The kinetic analysis was performed with MATLAB R2020a^50^ using a program developed by our group. This program has been successfully employed in several previous publications.^21,42,51,52^ The program can be categorized into two parts: simulation and kinetic fitting. For the simulation program, we employed the *ode 45* function solver and provided three types of inputs: the reaction mechanism with the given rate coefficients, the simulation time window, and the initial concentrations of species. The initial concentrations of CH_3_CHI_2_, CH_3_CHI, I, O_2_, He, and HCl were experimentally determined and used, while those of all other species were set as zero. This program simulates temporal profiles of each specific species. We then employed the *fmincon* function solver for the kinetic fitting. One or more experimental temporal profiles, expressed as *A*(*t*), *B*(*t*), *C*(*t*), etc., were used as inputs for either individual or simultaneous (global) fitting. Each profile was scaled by an arbitrary intensity factor, expressed as *f*_a_, *f*_b_, *f*_c_, etc., which were treated as free parameters. In the case involving overlapping signals *I*_multi_(*t*) from multiple species, an initial guess was constructed using a linear combination

$$I_{multi}\left(t\right)=f_{a}A\left(t\right)+f_{b}B\left(t\right)+f_{c}C\left(t\right)+...$$ 2

Each rate coefficient in the model may be treated as either a fixed parameter or a free parameter (with boundaries, if desired) in the fitting process. All free parameters, including intensity factors, were varied simultaneously to obtain the best fit with the experimental profile(s) using the least-squares method. To assess the sensitivity of a specific reaction, its rate coefficient was fixed at a specific value which was deviated from the original input value by a certain percentage, and the subsequently fitted rate coefficient of interest was then compared with the originally fitted value.

## Supplementary Material



## Notes

Details in kinetic fitting of *syn*-CH_3_CHOO + HCl (Note SA); spectral lines near 1280 cm^–1^ (Note SB); relative integrated absorbance (Note SC); derivation of *k*_*anti*_^I^ by method B (Note SD); derivation of *k*_*anti*_^I^ by method C (Note SE). Experimental conditions and fitted *k*_*syn*_^I^, kinetic model for *syn*-CH_3_CHOO + HCl, relative integrated absorbance (σ_*syn*_^883^/σ_*syn*_^1280^) of *syn*-CH_3_CHOO, relative integrated absorbance (σ_*syn*_^883^/σ_CEHP_^1280^) of *syn*-CH_3_CHOO and *anti*-CEHP, experimental conditions and fitted *k*_*anti*_^I^ (5 tables). Temporal profiles of *syn*-CH_3_CHOO reacting with HCl, comparison of experimental spectrum in different regions in various periods and with spectra simulated for *syn*-CH_3_CHOO, *anti*-CH_3_CHOO, CH_3_CHI_2_/O_2_, CH_3_CHI_2_/O_2_/HCl, temporal profiles of absorbance of various bands with or without HCl added, temporal profiles of bands of *syn*-CH_3_CHOO probed near 883 and 1280 cm^–1^ with or without HCl added, comparison of experimental profiles with those fitted for *syn*-CH_3_CHOO + *anti*-CEHP by assuming *k*_HCl_^*syn*^ = *k*_HCl_^*anti*^, derivation of temporal profiles of *anti*-CEHP and comparison with simulations, comparison of derived experimental profiles of *anti*-CEHP with simulations based on Method B, derivation of temporal profiles of *syn*- and *anti*-CH_3_CHOO from UV and IR probes in Method C (12 figures) (PDF)
