# Supplementary material for: Conformation-Specific Rate Coefficients of CH3CHOO + HCl Determined with Multiple IR/UV Absorption Probes
Source: J Phys Chem Lett. 2025 Jun 17;16(25):6455–62. doi: 10.1021/acs.jpclett.5c01353 (PMC12207663; doi:10.1021/acs.jpclett.5c01353)
Supplement: Supplementary file 1 [file jz5c01353_si_001.pdf]

Supporting Information

Conformation-Specific Rate Coefficients of CH_3CHOO + HCl Determined with Multiple IR/UV Absorption Probes

Tang-Yu Kao,[†] *Chen-An Chung,*[†] *and Yuan-Pern Lee*[†,‡*]

[†]Department of Applied Chemistry and Institute of Molecular Science, National Yang Ming Chiao Tung University, Hsinchu 300093, Taiwan

[‡]Center for Emergent Functional Matter Science, National Yang Ming Chiao Tung University, Hsinchu 300093, Taiwan

**E-mail: yplee@nycu.edu.tw* (Y.-P. Lee)

Table of Contents

Note SA. Details in kinetic fitting of *syn*-CH_3CHOO + HCl S1

Note SB. Spectral lines probed with a QCL near 1280 cm^{-1} S3

Note SC. Relative integrated absorbance S5

Note SD. Derivation of k_{anti}^{I} by method B S6

Note SE. Derivation of k_{anti}^{I} by method C S7

Table S1. Experimental conditions and the fitted first-order rate coefficients k_{syn}^{I} of *syn*-CH_3CHOO + HCl S9

Table S2. Kinetic model for fitting the reaction of *syn*-CH_3CHOO + HCl S10

Table S3. Determination of relative integrated absorbance ($\sigma_{syn}^{883}/\sigma_{syn}^{1280}$) of *syn*-$CH_3CHOO$ near 883 cm^{-1} and near 1280 cm^{-1} S11

Table S4. Determination of relative integrated absorbance ($\sigma_{syn}^{883}/\sigma_{CEHP}^{1280}$) of *syn*-$CH_3CHOO$ near 883 cm^{-1} and *anti*-CEHP near 1280 cm^{-1} S12

Table S5. Experimental conditions and fitted results of the reaction of *anti*-CH_3CHOO + HCl S13

Figure S1. Temporal profiles of *syn*-CH_3CHOO reacting with various initial concentrations of HCl, $[HCl]_0$, in experimental set 3 at 298 K S14

Figure S2. Comparison of experimental spectrum in region 1279.5−1280.8 cm^{-1} with spectra simulated for *syn*-CH_3CHOO and *anti*-CH_3CHOO S15

Figure S3. Comparison of spectra in region 1279.6−1280.5 cm^{-1} in various reaction periods in experiments of CH_3CHI_2/O_2 .... S16

Figure S4. Temporal profiles of absorbance of various bands when no HCl was added. S17

Figure S5. Comparison of spectra in region 1279.6−1280.2 cm^{-1} in various reaction periods in experiments with $CH_3CHI_2/O_2/HCl$. S18

Figure S6. Temporal profiles of absorbance of various bands when HCl was added. S19

Figure S7. Temporal profiles of bands of *syn*-CH_3CHOO probed near 883 and 1280 cm^{-1} under identical experimental conditions without adding HCl S20

Figure S8. Temporal profiles of bands probed near 883 and 1280 cm^{-1} under similar experimental conditions with HCl added S21

Figure S9. Comparison of temporal profiles probed near 1280 cm^{-1} with those of *syn*-CH_3CHOO + *anti*-CEHP fitted based on a kinetic model listed in Table 1 by assuming $k_{HCl}^{syn} = k_{HCl}^{anti}$ S22

Figure S10. Derivation of temporal profiles of *anti*-CEHP and comparison with simulations. S23
Figure S11. Comparison of derived temporal profiles of *anti*-CEHP in experimental set 2 with simulated profiles of *anti*-CEHP based on Method B S24
Figure S12. Derivation of temporal profiles of *syn*- and *anti*-CH_3CHOO from UV and IR probes in Method C S25
Supporting References S26

Note SA. Details in kinetic fitting of *syn*-CH_3CHOO + HCl

The experimental conditions for reactions of *syn*-CH_3CHOO + HCl are summarized in Table S1. We employed the previously reported kinetic model,[1] including three channels of the formation reaction, self-reactions of *syn*- and *anti*-CH_3CHOO, the cross-reaction between *syn*-CH_3CHOO and *anti*-CH_3CHOO, possible secondary reactions involving CH_3CHOO and CH_3CHIOO, and added the title reactions *syn*-CH_3CHOO + HCl and *anti*-CH_3CHOO + HCl along with the unimolecular decomposition of the reaction product, as listed in Table S2. The rate coefficient (k_{form}) of the formation reaction CH_3CHI + O_2 was set as 3.8×10^{-12} cm^3 $molecule^{-1}$ s^{-1}.[1] The branching ratio of the adduct CH_3CHIOO from CH_3CHI + O_2 was set as 0.14 according to Howes et al.,[2] and the branching ratio for *syn*-CH_3CHOO : *anti*-CH_3CHOO was set as 80 : 20.[1] For each formation channel, we allowed ±18% variation of the rate coefficients in fitting to account for the possible errors in these literature values. The rate coefficients for the self-reactions of *syn*-CH_3CHOO and *anti*-CH_3CHOO were set as k_{self}^{syn} = 1.4×10^{-10} cm^3 $molecule^{-1}$ s^{-1} and k_{self}^{anti} = 6.0×10^{-10} cm^3 $molecule^{-1}$ s^{-1}, as reported previously,[1] and was allowed ±20% and ±30% variations in fitting to account for their uncertainties. The rate coefficients for the cross-reactions between *syn*-CH_3CHOO and *anti*-CH_3CHOO was set as $k_{\mathrm{self}}^{cross}$ = 2.1×10^{-10} cm^3 $molecule^{-1}$ s^{-1} and allowed ±30% in fitting.[1] The reaction products for both *syn*- and *anti*-CH_3CHOO with HCl are both *anti*-CEHP, according to the report by Su and Lee.[3]

syn-CH_3CHOO + HCl $\rightarrow$ *anti*-CEHP (1a)

anti-CH_3CHOO + HCl $\rightarrow$ *anti*-CEHP (1b)

The rate coefficient of reaction 1b, k_{HCl}^{anti}, was set as 2.9×10^{-10} cm^3 $molecule^{-1}$ s^{-1}, as discussed in the main text, and allowed ±30% in fitting, whereas the pseudo-first-order rate coefficient of reaction 1a, $k_{syn}^{\mathrm{I}} = k_{\mathrm{HCl}}^{syn}\times[\mathrm{HCl}]_0$ was fitted. Other reactions, such as CH_3CHOO + I, CH_3CHIOO + I, and CH_3CHIOO + CH_3CHIOO, were also included, even though they play

minor roles in the fitting; their rate coefficients are unreported, so we used the same values as those corresponding to reactions involving CH_3CHOO.

In this model, we did not specify the products of the reaction of CH_3CHOO + I, which might produce $CH_3CHI + O_2$, CH_3CHIOO, and CH_3CHO + IO, as they have insignificant effects on k_{HCl}^{syn} and k_{HCl}^{anti}. The pressure dependence of these reactions was also not considered under our experimental conditions. The reaction CH_3CHOO + IO was not included because the effect of this reaction on k_{HCl}^{syn} and k_{HCl}^{anti} is expected to be small, as $[IO]_0$ is smaller than $[I]_0$.

Representative temporal profiles of concentrations of *syn*-CH_3CHOO at various initial concentrations of HCl (ranging from 6.6×10^{13} to 18.6×10^{13} molecule cm^{-3}, experimental set 3 in Table S1) and a total pressure of 8.0 Torr are shown in Figure S1. The initial concentration of *syn*-CH_3CHOO was estimated to be ~5.8×10^{12} molecule cm^{-3}. A summary of the fitted first-order rate coefficients (k_{syn}^{I}) for all experiments is presented in Table S1. Figure 1 illustrated the dependence of the fitted k_{syn}^{I} on $[HCl]_0$. From the slope of this plot , the bimolecular rate coefficient of *syn*-CH_3CHOO + HCl was determined to be $k_{HCl}^{syn} = (5.06 \pm 0.10)\times10^{-11}$ cm^3 $molecule^{-1}$ s^{-1}; the listed error represents one standard deviation in fitting. When the fitted line was constrained to pass through the origin, k_{HCl}^{syn} was calculated to be $(5.50 \pm 0.10)\times10^{-11}$ cm^3 $molecule^{-1}$ s^{-1}.

Considering the errors in the estimation of concentrations of *syn*-/*anti*-CH_3CHOO (±20%) that translate to an error of ±13% in k_{HCl}^{syn}, the error in k_{form} of the formation reaction (±18%) that translates to an error of ±9% in k_{HCl}^{syn}, the error in k_{self}^{syn} of the self-reactions of *syn*-CH_3CHOO (±20%) that translates to an error of ±9% in k_{HCl}^{syn}, the error in k_{self}^{cross} of the cross reaction of *syn*- and *anti*-CH_3CHOO (±30%) that translates to an error of ±4% in k_{HCl}^{syn}, the error in k_{self}^{anti} of the self-reaction of *anti*-CH_3CHOO (±30%) that translates to an error of ±2% in k_{HCl}^{syn}, the error in k_{HCl}^{anti} for the reaction of *anti*-CH_3CHOO + HCl (±30%) that translates to

an error of ±8% in k_{HCl}^{syn}, the fitting error of ±2%, and other typical errors in measurements of flow rates (3%), temperature (1%), and pressure (3%). The six side reactions, k_1–k_6, listed in Table 1 have negligible effect on the fitted first-order rate coefficient. We estimated the overall standard error to be ~21%. The bimolecular rate coefficient for *syn*-CH_3CHOO + HCl is thus reported as k_{HCl}^{syn} = (5.1 ± 1.1)×10^{-11} cm^3 $molecule^{-1}$ s^{-1}.

Note SB. Spectral lines probed with a QCL near 1280 cm^{-1}

We performed a high-resolution scan during photolysis of a CH_3CHI_2/O_2 mixture (without HCl) in region 1279.6−1280.5 cm^{-1}, and compared the observed spectrum with spectral simulations in Figure S2. The simulation with PGOPHER[4] incorporated the experimental rotational constants of ν_7 reported by Nakajima et al. as A'' =0.5866, B'' = 0.2379, and C'' = 0.1744 for *syn*-CH_3CHOO[5] and A'' = 1.6176, B'' = 0.1479, and C'' = 0.1390 for *anti*-CH_3CHOO.[6] The calculated ratio of rotational constants between the vibrationally excited states (v = 1) and the ground state (v = 0) was adapted from Lin et al.,[7] with values of A'/A'' = 0.9982, B'/B'' = 0.9981, and C'/C'' = 0.9980 for *syn*-CH_3CHOO, and A'/A'' = 0.9978, B'/B'' = 1.0000, and C'/C'' = 1.0000 for *anti*-CH_3CHOO; these values were calculated using the MULTIMODE method. The *a*/*b* type ratio was set as 0.42/0.58 for *syn*-CH_3CHOO and 0.59/0.41 for *anti*-CH_3CHOO, according to the fitted results from Lin et al.[7] The simulation parameters were set as T = 298 K, J_{max} = 200, and Gaussian linewidth = 0.003 cm^{-1}. The observed spectral bands have no clear one-to-one correspondence with the simulated spectral bands; the spectral assignment is hence extremely challenging. It is likely that interactions of overtone ($2\nu_{11}$) and combination bands (such as $\nu_9 + \nu_{12}$) significantly perturbed the ν_7 band.

In principle, both *syn*-CH_3CHOO and *anti*-CH_3CHOO are produced initially and *anti*-CH_3CHOO is expected to decay faster than *syn*-CH_3CHOO because of the difference in rate coefficients of self-reactions.[1] We compared spectra recorded at 30, 90, and 200 μs with that recorded at 45 μs in Figures S3a −S3c, respectively. In each frame, bands marked with blue

asterisks are normalized. Three types of bands, with intensities (relative to those marked with blue asterisks) decreasing, increasing, and remaining constant with time, were identified, as marked with purple circles, a green triangle, and blue asterisks, respectively. One expects that, if those with blue asterisks are associated with *syn*-CH_3CHOO, then those marked with purple circles are associated with *anti*-CH_3CHOO, which decays faster than *syn*-CH_3CHOO, and those with green triangle are associated with stable products. We then integrated bands marked with purple circles (1280.08−1280.09 and 1280.14−1280.15 cm^{-1}), green triangle (1280.00−1280.01 cm^{-1}), and blue asterisks (1279.93−1279.95, 1279.87−1279.89, and 1279.80−1279.82 cm^{-1}) to obtain their temporal behavior, as presented in Figure S4a, and compared with that of *syn*-CH_3CHOO obtained by probing regions 883.105−883.135 and 883.148−883.185 cm^{-1} (red squares) and a simulation (black line) for *syn*-CH_3CHOO using the model listed in Table S2 (without reactions with HCl). The temporal behavior of the bands marked with blue asterisks agree with the experimental (probed near 883 cm^{-1}) and the simulated results of *syn*-CH_3CHOO. In contrast, the bands marked with purple circles showed an initial rapid decay as expected for *anti*-CH_3CHOO (Figure S4b), but they were interfered with by a slow component (Figure S4a) at a later period, indicating that other species also contributed to this band. These bands hence cannot be used to monitor *anti*-CH_3CHOO. The band marked with a green triangle showed a slower decay, indicating that it might have contributions from a stable product.

Similarly, we performed a high-resolution scan during photolysis of a $CH_3CHI_2/O_2/HCl$ mixture in region 1279.7−1280.2 cm^{-1}. We compared spectra recorded at 45, 60, and 300 μs with that recorded at 30 μs in Figures S5a−S5c, respectively. At 300 μs, one would expect that all bands of CH_3CHOO diminished because they have reacted with HCl. However, the green trace (300 μs) in Figure S5c shows a non-zero continuous absorption with a band marked with green triangle. On inspection of the literature IR spectrum of the reaction product of CH_3CHOO + HCl, *anti*-CEHP ($CH_3CHClOOH$), a band (marked B_4) lies in region 1250−1290 cm^{-1} with

a Q-band at 1271.8 cm^{-1};[3] this band overlaps with the ν_7 band of CH_3CHOO near 1280 cm^{-1}. The continuous absorption (green trace in Figure S5c) is likely due to *anti*-CEHP. Furthermore, the regions marked with purple circles diminished near 60 μs into a continuous background, likely because of the rapid reaction of *anti*-CH_3CHOO with HCl in the early reaction stage. We then integrated absorbance over spectral regions marked with purple circles, blue asterisks, and the green triangle to obtain their temporal behavior, as compared in Figure S6 with that obtained in regions 883.105−883.135 and 883.148−883.185 cm^{-1} (red squares) and a simulation for *syn*-CH_3CHOO (black line) and *syn*-CH_3CHOO + *anti*-CEHP (golden line) using the model listed in Table 1. The temporal behavior of the bands marked with blue asterisks (blue marks) agrees with the simulated results of *syn*-CH_3CHOO + *anti*-CEHP, whereas the experimental result probed near 883 cm^{-1} (red marks) agree well with the simulated results of *syn*-CH_3CHOO. In contrast, the temporal profiles of bands marked with purple circles and the green triangle exhibit additional contribution from other species, so that they do not agree with the simulated temporal profiles.

Although definitive spectral assignments are unlikely to be derived from the spectrum in Figure S2, we could still find bands of those marked with blue asterisks and purple circles to correlate with bands in the simulated spectra of *syn*-CH_3CHOO and *anti*-CH_3CHOO, respectively, as indicated with dashed lines. The band origins of *syn*-CH_3CHOO and *anti*-CH_3CHOO were shifted to achieve such correspondence. Even though these shifts are somewhat arbitrary, such a correspondence supports that those bands marked with blue asterisks are due to *syn*-CH_3CHOO. Although bands marked with purple circles appear to correspond to *anti*-CH_3CHOO, they are weak and have additional contributions from other species. We hence are unable to probe these bands and attribute them to only *anti*-CH_3CHOO.

Note SC. Relative integrated absorbance

We determined the relative integrated absorbance for bands of *syn*-CH_3CHOO near 883 cm^{-1} and those near 1280 cm^{-1} (the integrated regions are listed in Note SB) by measuring the temporal profiles of integrated absorbance using these two QCL under identical experimental conditions (without HCl) and normalizing them to derive the ratios of integrated absorbance ($\sigma_{syn}^{883}/\sigma_{syn}^{1280}$) in these two regions, as shown in Figure S7. This ratio was derived to be 6.8 ± 0.3 from 5 experiments, as listed in Table S3. This allows us to convert temporal profiles of *syn*-CH_3CHOO probed near 883 cm^{-1}, which is free from interference by absorption of *anti*-CEHP, to those near 1280 cm^{-1} by dividing the ratio of $\sigma_{syn}^{883}/\sigma_{syn}^{1280}$.

Similarly, as shown in Figures S8, by measuring temporal profiles of integrated absorbance of *anti*-CEHP near 1280 cm^{-1} (red) at the later stage under conditions of excessive HCl (so that all CH_3CHOO were converted to CEHP) and those of *syn*-CH_3CHOO near 883 cm^{-1} in the initial stage (black, without HCl added) and comparing them, we derived the ratios of integrated absorbance ($\sigma_{syn}^{883}/\sigma_{\mathrm{CEHP}}^{1280}$) between *syn*-$CH_3CHOO$ near 883 cm^{-1} and *anti*-CEHP near 1280 cm^{-1}. The initial integrated absorbance of *syn*-CH_3CHOO was derived by extrapolating the $1/A_{883}$ to reaction time zero to account for the decay due to self-reaction. The integrated absorbance of *anti*-CEHP was obtained on extrapolating the red trace to time zero to account for a small slow decay due to pumping. The ratio $\sigma_{syn}^{883}/\sigma_{\mathrm{CEHP}}^{1280}$ was estimated to be 7.3 ± 0.7 from 5 experiments, as summarized in Table S4.

The ratio of integrated absorbance between *syn*-CH_3CHOO and *anti*-CEHP near 1280 cm^{-1}, $\sigma_{syn}^{1280}/\sigma_{\mathrm{CEHP}}^{1280}$, was consequently derived from $\sigma_{syn}^{883}/\sigma_{syn}^{1280}$ (= 6.8 ± 0.3) and $\sigma_{syn}^{883}/\sigma_{\mathrm{CEHP}}^{1280}$ (= 7.3 ± 0.7) to be 1.06 ± 0.11, and employed to compare with the conversion factor ratios of $f_{\mathrm{CI}}/f_{\mathrm{CEHP}}$ derived in the model fitting (method A) of the combined signal of *syn*-CH_3CHOO and *anti*-CEHP in IR measurements near 1280 cm^{-1}.

Note SD. Derivation of k_{anti}^{I} by method B

In Method B, we derived temporal profiles of *anti*-CEHP and fitted them with the kinetic model to derive k_{anti}^{I}. Representative temporal profiles probed near 1280 cm^{-1} (black squares) are shown in Figure S10 for experiments with $[HCl]_0$ = 2.4 mTorr (frame a) and $[HCl]_0$ = 17.1 mTorr (frame b). The *syn*-CH_3CHOO profiles probed near 883 cm^{-1} were converted to the *syn*-CH_3CHOO profiles near 1280 cm^{-1} (red circles) by dividing them with $\sigma_{syn}^{883}/\sigma_{syn}^{1280}$. Subtracting the red profiles from the black profiles yielded a profile presented with blue triangles, corresponding to the absorbance of the product, *anti*-CEHP. The initial concentration of *syn*-CH_3CHOO, [*syn*-$CH_3CHOO]_0$, was estimated from $[CH_3CHI]_0$, probed with the 286-nm LED light, and the literature value of the branching ratio, 0.86×0.80, of *syn*-CH_3CHOO, which is consistent with the absolute concentration of [*syn*-$CH_3CHOO]_0$ determined using the 335-nm UV probe and the branching ratio 0.86. The pink lines in Figure S10 represent simulated profiles of *anti*-CEHP using Method A after conversion of concentration to absorbance; the agreements between the blue triangles and the pink traces are satisfactory, indicating that the derivation of temporal profiles of *anti*-CEHP is reliable.

By applying the ratio $\sigma_{syn}^{1280}/\sigma_{CEHP}^{1280}$ (1.06 ± 0.11), we converted the absorbance temporal profile of *anti*-CEHP to absolute concentration of [*anti*-CEHP]$_t$ by the following equation.

$$[anti\text{-CEHP}]/I_{CEHP}^{1280} = ([syn\text{-CH}_3\text{CHOO}]/I_{syn}^{1280})(\sigma_{syn}^{1280}/\sigma_{CEHP}^{1280}) \quad (3)$$

in which I_{CEHP}^{1280} and I_{syn}^{1280} represent the integrated absorbance of *anti*-CEHP and *syn*-CH_3CHOO near 1280 cm^{-1}. The blue traces obtained in Figure S10 were fitted with the kinetic model listed in Table 1. The derived temporal profiles of [*anti*-CEHP] (symbols) for experimental set 2 and the simulated profiles (lines) according to kinetic modeling are presented in Figure S11. The k_{anti}^{I} values derived by Method B for experimental set 2 are listed in Table S5 to compare with those derived by Method A. The average absolute deviation is 9.5 ± 12.2%, reflecting the possible errors in fitting with these two methods.

Note SE. Derivation of k_{anti}^{I} by method C

In Method C, we compared the UV profile probed at 335 nm (for both *syn*- and *anti*-CH_3CHOO) with the IR profile probed near 883 cm^{-1} (for *syn*-CH_3CHOO) by scaling and matching the slow decay components, representing only *syn*-CH_3CHOO, in the later reaction period. Subtracting these two traces allowed us to obtain the temporal profiles of *anti*-CH_3CHOO for kinetic modeling. A representative set of UV-probed and IR-probed traces normalized at the maxima are shown in Figure S12a; the decay of the UV (red) trace is slightly more rapid than that of the IR (black) trace. Figure S12b shows the two traces with the decays in the later period (after ~0.3 ms) scaled; an extra component in the UV profile in the initial stage was clearly visible. This component, shown as the blue trace in Figure S12b, corresponds to the UV absorption of *anti*-CH_3CHOO. After conversion from absorbance to concentrations by taking account of the UV cross sections, the rate coefficient k_{anti}^{I} was derived by fitting the profile of *anti*-CH_3CHOO (blue) with the kinetic model listed in Table 1. The results for experimental set 2 are compared with those derived from Methods A and B in Table S5. The average absolute deviation from those derived with Method A is 21 ± 17%, reflecting that this method is associated with larger errors because of the small component derived by subtracting two large values. Nevertheless, the agreement is still within the uncertainties (±27%) discussed in the main text, supporting that *anti*-CH_3CHOO indeed existed in the system and has a greater reactivity toward HCl.

Table S1. Experimental conditions and the fitted first-order rate coefficients k_{syn}^{I} of *syn*-CH_3CHOO + HCl

set	expt.	P_T /Torr	$[CH_3CHI_2]_0$ /$10^{14\ b}$	$[CH_3CHI]_0$ /$10^{12\ b}$	$[O_2]$ /$10^{16\ b}$	[He] /$10^{16\ b}$	$[HCl]_0$ /$10^{13\ b}$	$k_{syn}^{I\ a}$ /10^3 s^{-1}
1	1	4.0	3.7	8.3	13.1	0	9.6	5.7
	2	4.0	3.7	8.3	13.0	0	13.4	8.5
	3	4.0	3.7	8.4	12.8	0	16.0	10.2
2	4	4.0	7.6	16.9	12.8	0	8.8	5.6
	5	4.0	7.6	16.6	12.8	0	12.2	7.9
	6	4.0	7.6	16.7	12.8	0	18.3	10.0
3	7	8.0	3.7	8.1	25.7	0	6.6	4.3
	8	8.0	3.7	8.4	25.7	0	12.6	8.2
	9	8.0	3.7	8.4	25.7	0	18.6	11.0
4	10	8.0	7.6	16.6	26.1	0	8.6	6.2
	11	8.0	7.6	16.6	25.7	0	14.6	9.3
	12	8.0	7.6	16.9	25.7	0	22.6	13.0
5	13	10.0	11.8	39.8	6.9	25.3	32.7	17.8
	14	10.0	8.5	28.5	2.1	30.1	33.7	17.9
	15	10.0	9.4	31.7	2.1	30.1	54.7	28.0
6	16	16.0	17.2	54.8	3.4	48.1	35.6	19.7
	17	16.0	17.2	55.1	3.4	48.1	55.0	31.5

[a]Fitted from temporal profiles of *syn*-CH_3CHOO according to the model listed in Table 1. [b]In molecule cm^{-3}.

Table S2. Kinetic model for fitting the reaction of *syn*-CH_3CHOO + HCl

reaction		rate coefficient[a,b]	reference
$CH_3CHI + O_2 \rightarrow$ *syn*-CH_3CHOO + I	k_{form}^{a}	$x \times y \times 3.8{\times}10^{-12}$ (±18%) $x = 0.86$; $y = 0.80$	[1], [8]
$CH_3CHI + O_2 \rightarrow$ *anti*-CH_3CHOO + I	k_{form}^{b}	$x \times (1 - y) \times 3.8{\times}10^{-12}$ (±18%)	[1], [8]
$CH_3CHI + O_2 \rightarrow CH_3CHIOO$	k_{form}^{c}	$(1 - x) \times 3.8{\times}10^{-12}$ (±18%)	[2]
2 *syn*-$CH_3CHOO \rightarrow 2\ CH_3CHO + O_2$	k_{self}^{syn}	$1.4{\times}10^{-10}$ (±20%)	[1]
2 *anti*-$CH_3CHOO \rightarrow 2\ CH_3CHO + O_2$	k_{self}^{anti}	$6{\times}10^{-10}$ (±30%)	[1]
syn-CH_3CHOO + *anti*-CH_3CHOO $\rightarrow 2\ CH_3CHO + O_2$	k_{self}^{cross}	$2.1{\times}10^{-10}$ (±30%)	[1]
syn-CH_3CHOO + HCl → *anti*-CEHP	k_{HCl}^{syn}	fitted	
anti-CH_3CHOO + HCl → *anti*-CEHP	k_{HCl}^{anti}	$2.9{\times}10^{-10}$ (± 30%)	This work
syn-CH_3CHOO + I → products	k_1	9.0×10^{-12}	[9]
anti-CH_3CHOO + I → products	k_2	9.0×10^{-12}	[9]
$CH_3CHIOO + I \rightarrow CH_3CHIO + IO$	k_3	3.5×10^{-11}	[9]
$2\ CH_3CHIOO \rightarrow 2\ ICH_3CHO + O_2$	k_4	9.0×10^{-11}	[9]
$CH_3CHIO \rightarrow CH_3CHO + I$	k_5	$10^6\ s^{-1}$	[10]
$2\ IO \rightarrow I_2 + O_2$	k_6	9.9×10^{-11}	[9]

[a]Listed second-order rate coefficients are in cm^3 $molecule^{-1}$ s^{-1}, unless noted. [b]*x* is the branching ratio of *syn*-CH_3CHOO and *anti*-CH_3CHOO in total products and *y* is the branching ratio of *syn*-CH_3CHOO in total CH_3CHOO.

Table S3. Determination of relative integrated absorbance ($\sigma_{syn}^{883}/\sigma_{syn}^{1280}$) of *syn*-$CH_3CHOO$ near 883 cm^{-1} and near 1280 cm^{-1}

expt.	P_T /Torr	$[CH_3CHI_2]_0$ /$10^{15\ a}$	$[CH_3CHI]_0$ /$10^{13\ a}$	$[O_2]$ /$10^{17\ a}$	$[HCl]_0$ /$10^{13\ a}$	$\sigma_{syn}^{883}/\sigma_{syn}^{1280\ b}$
1	10.0	1.4	3.8	3.2	0	6.8
2	10.0	1.6	3.8	3.2	0	7.2
3	10.0	2.1	5.9	3.1	0	7.1
4	8.0	1.0	2.6	2.6	0	6.4
5	8.0	1.3	3.5	2.6	0	6.6
					Average	6.8 ± 0.3

[a]In molecule cm^{-3}. [b]Probed regions are 883.105−883.135 and 883.148−883.185 cm^{-1} for σ_{syn}^{883} and 1279.93−1279.95, 1279.87−1279.89, and 1279.80−1279.82 cm^{-1} for σ_{syn}^{1280}.

Table S4. Determination of relative integrated absorbance ($\sigma_{syn}^{883}/\sigma_{CEHP}^{1280}$) of *syn*-$CH_3CHOO$ near 883 cm^{-1} and *anti*-CEHP near 1280 cm^{-1}

expt.	P_T /Torr	$[CH_3CHI_2]_0$ /$10^{14\ a}$	$[CH_3CHI]_0$ /$10^{13\ a}$	$[O_2]$ /$10^{16\ a}$	[He] /$10^{16\ a}$	$[HCl]_0$ /$10^{14\ a,b}$	$\sigma_{syn}^{883}/\sigma_{CEHP}^{1280\ c}$
1	10	11.8	4.0	6.9	25.3	5.5	7.1
2	10	8.5	2.9	2.1	30.1	3.4	7.4
3	10	9.1	3.0	2.1	30.1	5.2	6.4
4	16	17.2	5.5	3.4	48.1	5.5	8.2
5	16	14.7	4.7	3.4	48.1	9.1	7.6
						Average	7.3 ± 0.7

[a]In molecule cm^{-3}. [b]Listed $[HCl]_0$ only represent that used in determining *anti*-CEHP. [c]Probed regions are 883.105−883.135 and 883.148−883.185 cm^{-1} for σ_{syn}^{883} and 1279.93−1279.95, 1279.87−1279.89, and 1279.80−1279.82 cm^{-1} for σ_{CEHP}^{1280}.

Table S5. Experimental conditions and fitted results of the reaction of *anti*-CH_3CHOO + HCl

set	expt.	P_T /Torr	$[CH_3CHI_2]_0$ /$10^{14\ d}$	$[CH_3CHI]_0$ /$10^{13\ d}$	$[O_2]$ /$10^{16\ d}$	[He] /$10^{16\ d}$	$[HCl]_0$ /$10^{14\ d}$	f_{CI}/f_{CEHP}	k_{anti}^{I} A[a] /10^4 s^{-1}	k_{anti}^{I} B[b] /10^4 s^{-1}	k_{anti}^{I} C[c] /10^4 s^{-1}	k_{loss} / s^{-1}
1	1	10	9.1	3.1	6.9	25.3	0.8	0.96	3.2			125
	2	10	12.4	4.2	6.9	25.3	1.4	0.91	3.8			130
	3	10	12.4	4.2	6.9	25.3	2.2	1.08	5.5			119
	4	10	11.8	4.0	6.9	25.3	3.3	1.07	8.8			133
	5	10	11.8	4.0	6.9	25.3	5.5	1.02	16.8			126
2	6	10	6.9	2.4	2.1	30.1	0.4	0.91	1.9	2.1	1.5	120
	7	10	6.9	2.4	2.1	30.1	0.8	0.88	2.9	3.0	3.0	129
	8	10	8.5	2.9	2.1	30.1	1.4	0.95	4.1	5.9	5.1	125
	9	10	8.5	2.9	2.1	30.1	3.4	1.03	8.0	8.0	11.7	123
	10	10	9.4	3.2	2.1	30.1	5.5	0.92	17.8	17.1	19.9	128
3	11	16	12.4	4.1	3.4	48.1	0.6	0.81	2.0			136
	12	16	17.2	5.5	3.4	48.1	1.3	0.98	3.7			118
	13	16	17.2	5.5	3.4	48.0	2.2	0.96	8.5			139
	14	16	17.2	5.5	3.4	48.1	3.6	1.19	10.1			120
	15	16	17.2	5.5	3.4	48.1	5.5	0.99	14.0			132
	16	16	14.7	4.7	3.4	48.1	9.1	1.09	27.9			126
average								0.99				127
deviation								± 0.10				± 6

[a]Fitted from temporal profiles of *syn*-CH_3CHOO + *anti*-CEHP probed near 1280 cm^{-1} according to the model listed in Table S2 (Method A). [b]Fitted from temporal profiles of *anti*-CEHP (Method B). [c]Fitted from temporal profiles of *anti*-CH_3CHOO (Method C). [d]In molecule cm^{-3}.

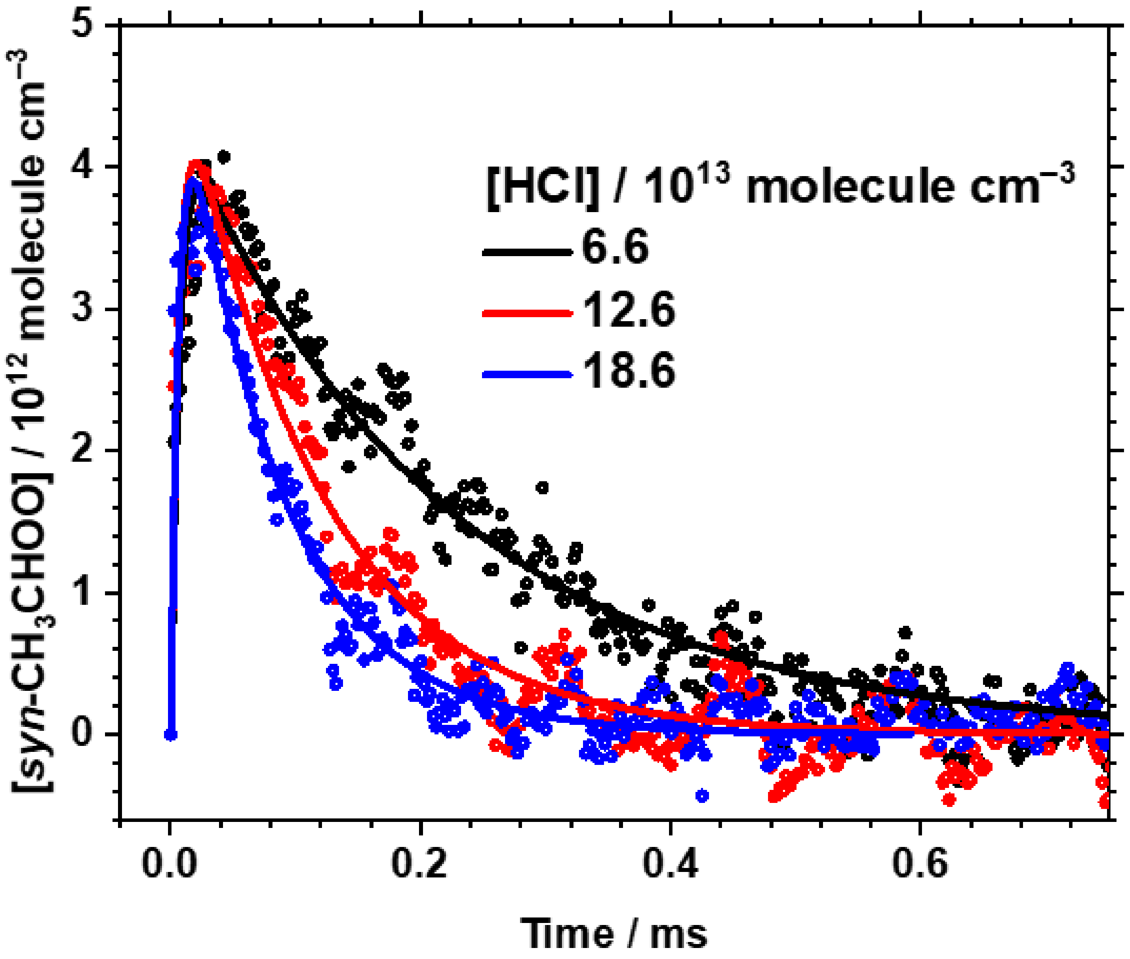

Figure S1. Temporal profiles of *syn*-CH_3CHOO reacting with various initial concentrations of HCl, $[HCl]_0$, in experimental set 3 at 298 K. Integrated absorbance in regions 883.105−883.135 and 883.148−883.185 cm^{-1} was probed. $[CH_3CHI_2]_0/[O_2]$ = 0.012/8.0, P_T = 8.0 Torr, and $[HCl]_0$ = (2.0–5.8) mTorr or (6.6−18.6)×10^{13} molecule cm^{-3}. Symbols represent experimental data and solid lines represent temporal profiles fitted with the kinetic model listed in Table 1.

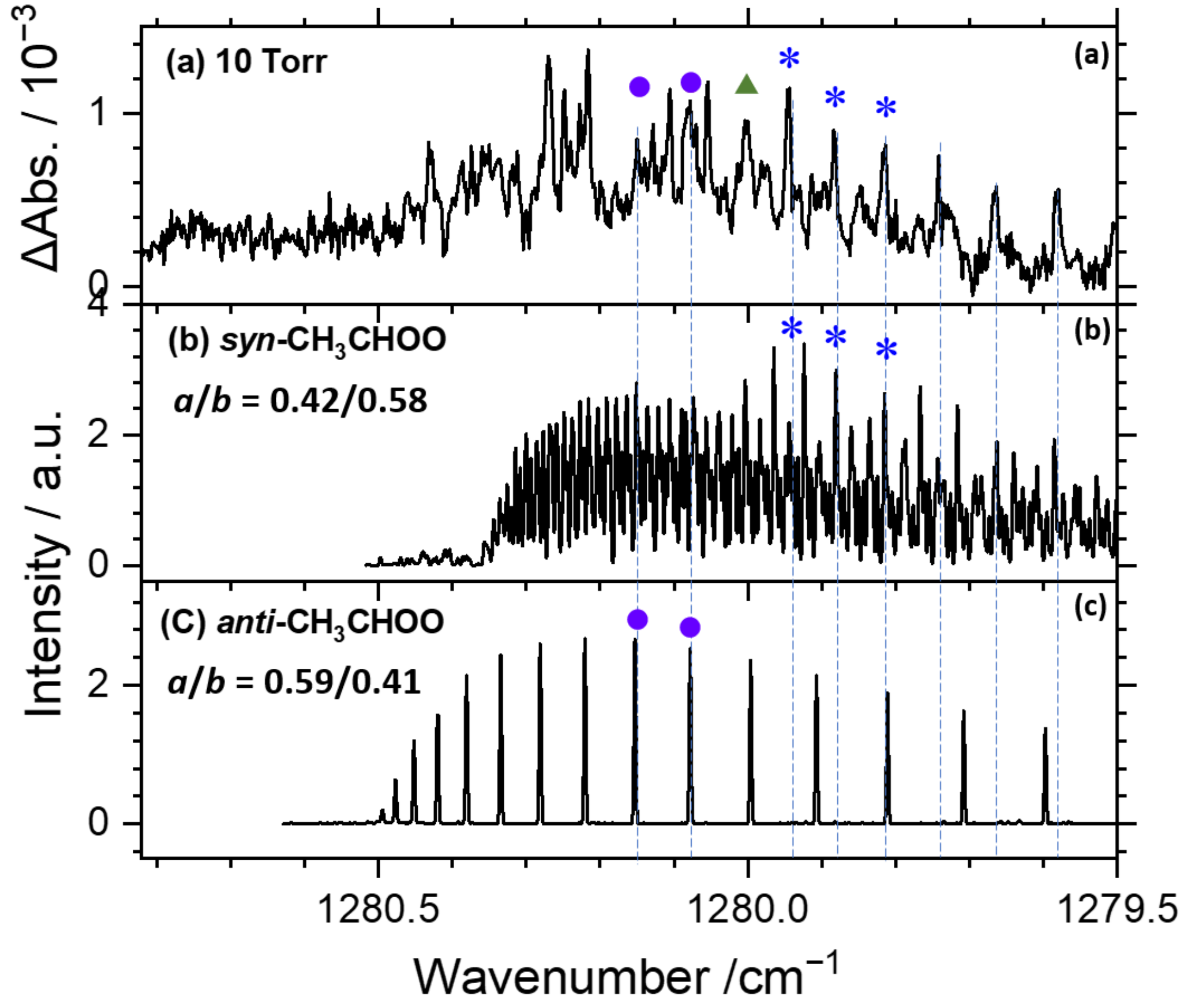

Figure S2. Comparison of experimental spectrum in region 1279.5−1280.8 cm^{-1} with spectra simulated for *syn*-CH_3CHOO and *anti*-CH_3CHOO. (a) Experimental spectrum recorded at 10 Torr; (b) Simulated spectrum of *syn*-CH_3CHOO; (c) Simulated spectrum of *anti*-CH_3CHOO. The spectra were simulated with PGopher according to molecular parameters predicted with the B3LYP/aug-cc-pVTZ method and experimental rotational constants of the ground state; see text. Lines marked with blue asterisks (*) are associated with *syn*-CH_3CHOO, whereas those marked with purple circles (●) might be associated with *anti*-CH_3CHOO. The line marked with a green triangle (▲) might be associated with a stable end product.

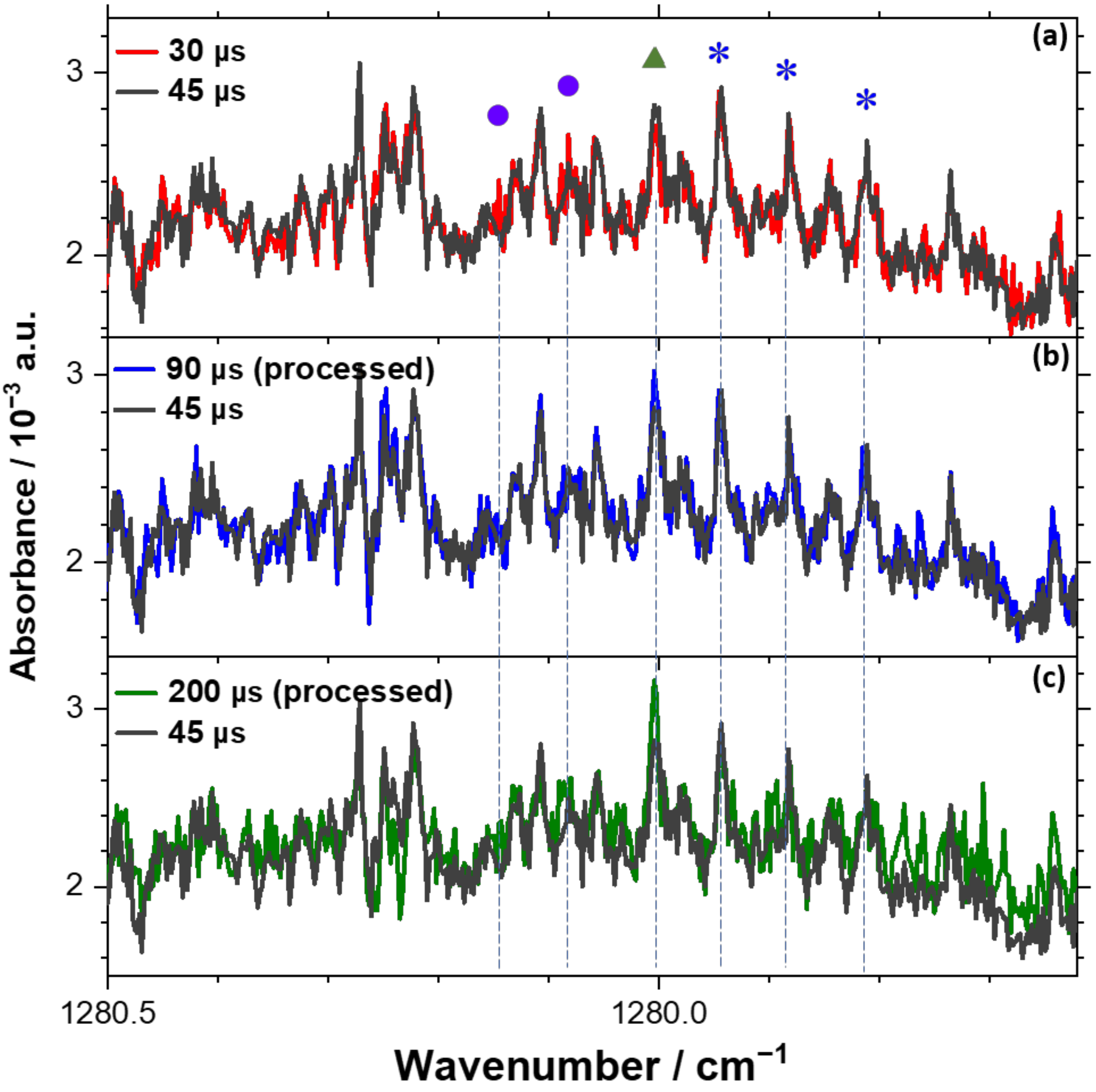

Figure S3. Comparison of spectra in region 1279.6−1280.5 cm^{-1} in various reaction periods in experiments of CH_3CHI_2/O_2. $[CH_3CHI_2]_0$ = 0.6 Torr, P_T = 10.0 Torr, and T = 298 K. The spectra are compared with that recorded at 45 μs (gray). (a) 30 μs, (b) 90 μs, (c) 200 μs. In each frame, bands marked with blue asterisks (*) are normalized. Bands marked with purple circles (●) showed decreasing trend, whereas a band marked with green triangle (▲) showed increasing trend.

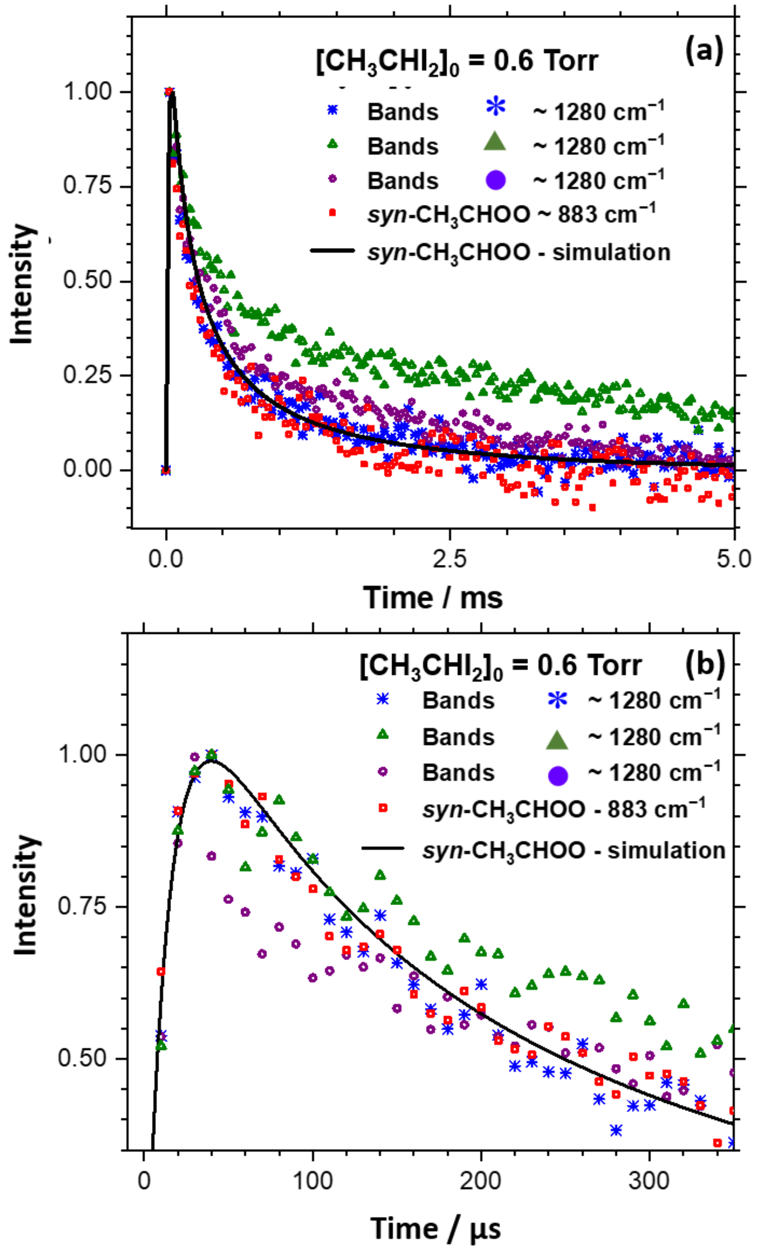

Figure S4. Temporal profiles of absorbance of various bands when no HCl was added. (a) 0−5 ms; (b) 0−350 µs. $[CH_3CHI_2]_0$ = 0.6 Torr, P_T = 10.0 Torr, and T = 298 K. ○ (purple circle): 1280.08−1280.09 and 1280.14−1280.15 cm^{-1}; △ (green triangle): 1280.00−1280.01 cm^{-1}; * (blue asterisk): 1279.93−1279.95 cm^{-1}, 1279.87−1279.89 cm^{-1}, and 1279.80−1279.82 cm^{-1}. These profiles are compared with the temporal profiles of *syn*-CH_3CHOO recorded in regions 883.105−883.135 cm^{-1} and 883.148−883.185 cm^{-1} (□, red squares) and a simulated profile for *syn*-CH_3CHOO (black line) according to the model in Table 1.

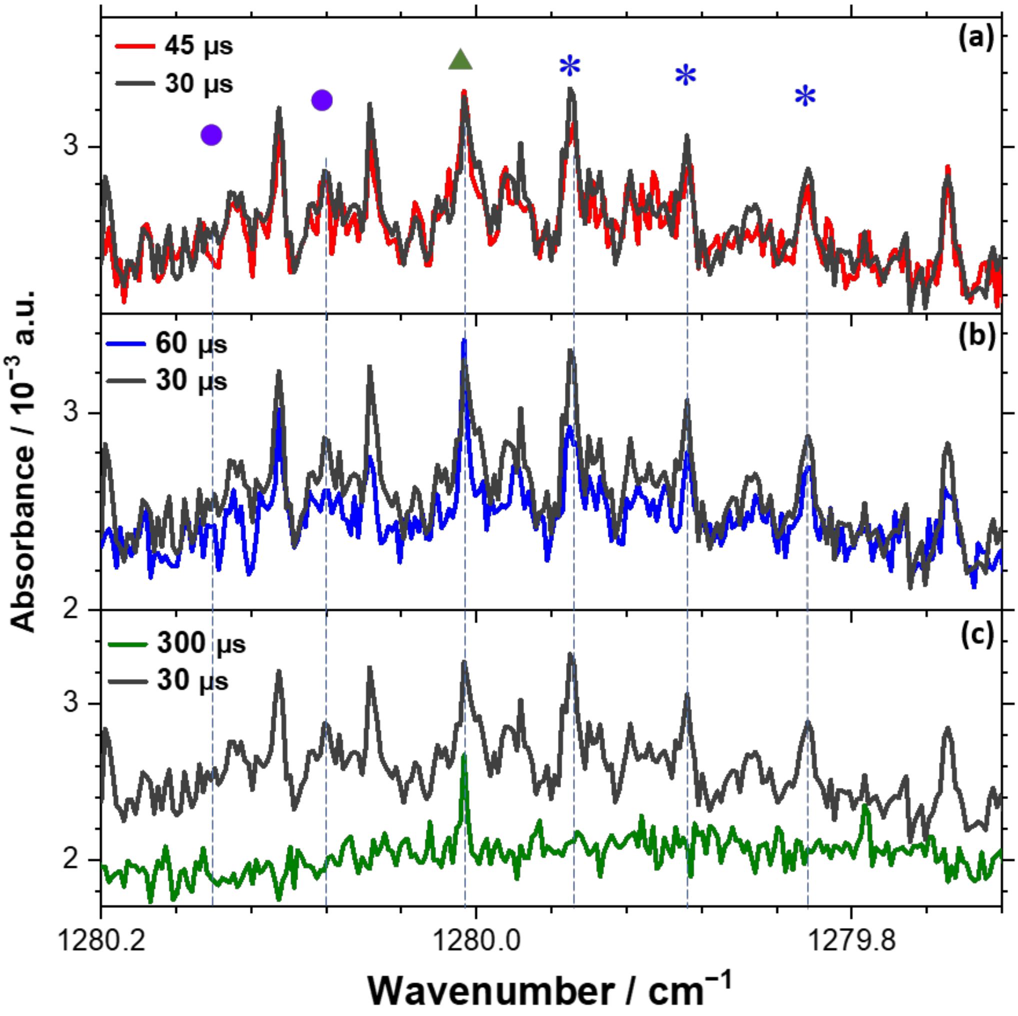

Figure S5. Comparison of spectra in region 1279.6−1280.2 cm^{-1} in various reaction periods in experiments with $CH_3CHI_2/O_2/HCl$. $[CH_3CHI_2]_0$ = 0.6 Torr, $[HCl]_0$ = 8.5 mTorr, P_T = 10.0 Torr, and T = 298 K. The spectrum recorded at 30 μs (black) is used for comparison. (a) 45 μs; (b) 60 μs; (c) 300 μs. Bands marked with purple circles (●) decreased to a continuous background after 60 μs. Most other bands decreased to a continuous background after 300 μs (frame c, green trace). The band marked with green triangle (▲) persists.

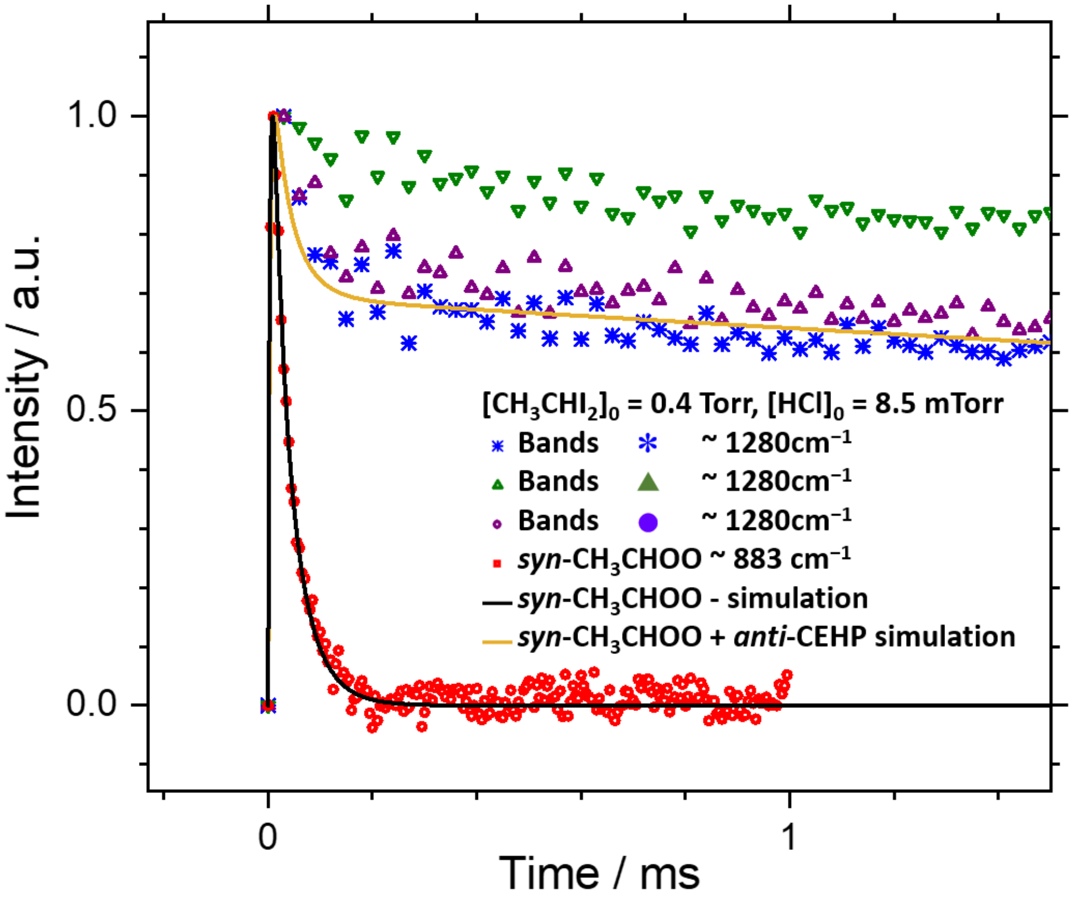

Figure S6. Temporal profiles of absorbance of various bands when HCl was added. $[CH_3CHI_2]_0$ = 0.6 Torr, $[HCl]_0$ = 8.5 mTorr, P_T = 10.0 Torr, and T = 298 K. ○ (purple circles): 1280.08−1280.09 and 1280.14−1280.15 cm^{-1}; △ (green triangle): 1280.00−1280.01 cm^{-1}; * (blue asterisks): 1279.93−1279.95 cm^{-1}, 1279.87−1279.89 cm^{-1}, and 1279.80−1279.82 cm^{-1}. These profiles are compared with the temporal profiles of *syn*-CH_3CHOO recorded in regions 883.105−883.135 and 883.148−883.185 cm^{-1} (red squares, □) and a simulation profile for *syn*-CH_3CHOO (black line) and *syn*-CH_3CHOO + *anti*-CEHP (gold line) according to the model in Table 1.

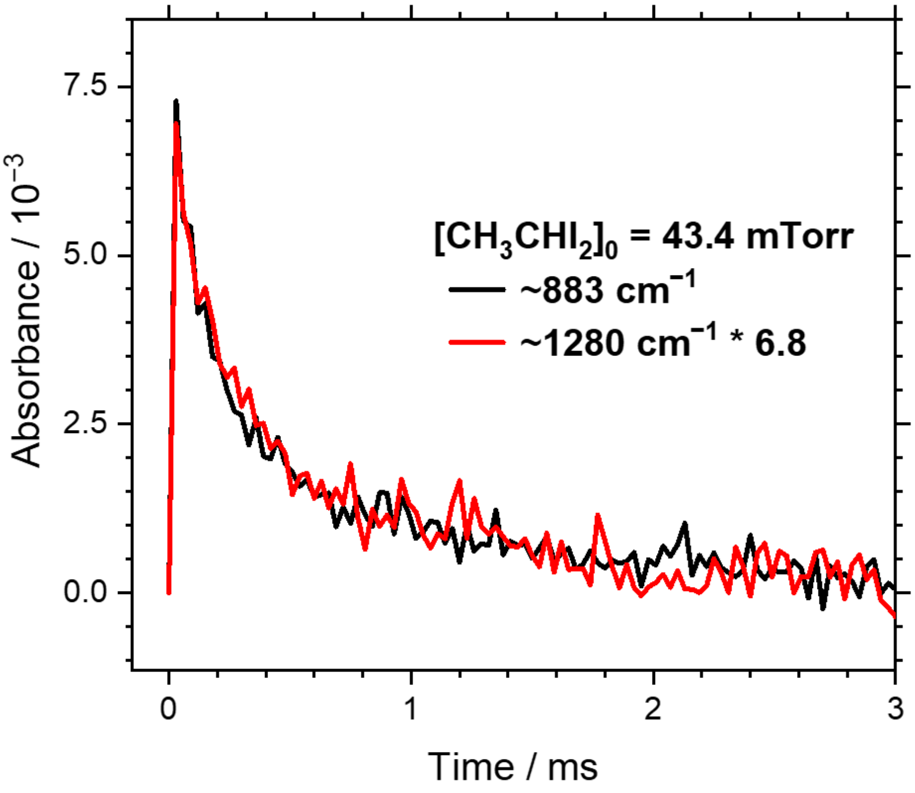

Figure S7. Temporal profiles of bands of *syn*-CH_3CHOO probed near 883 and 1280 cm^{-1} under identical experimental conditions without adding HCl. [CH_3CHI_2]$_0$ = 0.4 Torr, P_T = 10.0 Torr, and T = 298 K. The red trace (probed near 1280 cm^{-1}) was multiplied by a factor of 6.8 to match the black trace (probed near 883 cm^{-1}). The integrated absorbance ratio ($\sigma_{syn}^{883}/\sigma_{syn}^{1280}$) was determined to be 6.8 ± 0.3 based on five experiments listed in Table S3.

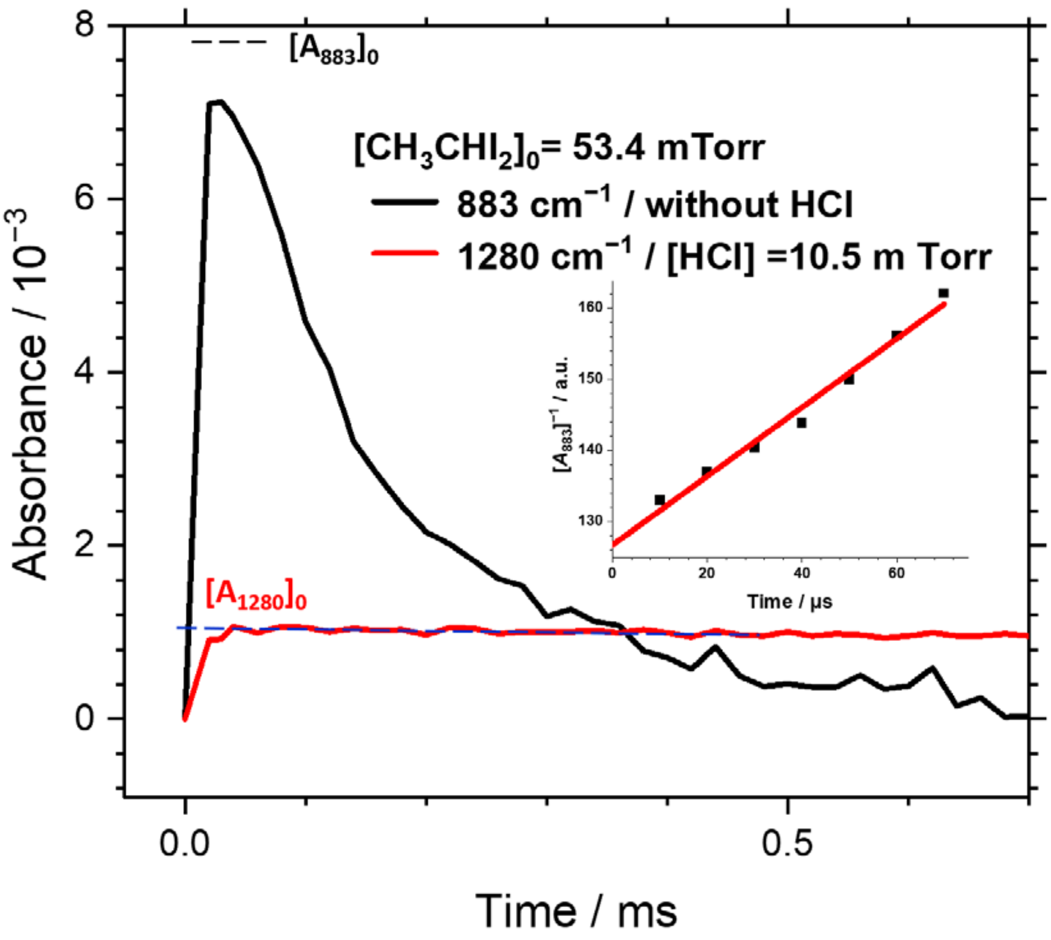

Figure S8. Temporal profiles of bands probed near 883 and 1280 cm^{-1} under similar experimental conditions with HCl added. $[CH_3CHI_2]_0$ = 53.4 mTorr, P_T = 10.0 Torr, T = 298 K, $[O_2]$ = 1.1 Torr, $[HCl]_0$ = 10.5 mTorr. The red trace represents absorbance probed near 1280 cm^{-1} (with excessive HCl added), whereas the black trace was probed near 883 cm^{-1} (without HCl added). The initial absorbance of *syn*-CH_3CHOO was derived by extrapolating the $1/A_{883}$ to reaction time zero and compared with the final absorbance of *anti*-CEHP, obtained on extrapolating the red trace to time zero to account for a small slow decay. The integrated absrobance ratio ($\sigma_{syn}^{883}/\sigma_{CEHP}^{1280}$) was estimated to be 7.3 ± 0.7 based on 5 experiments listed in Table S4.

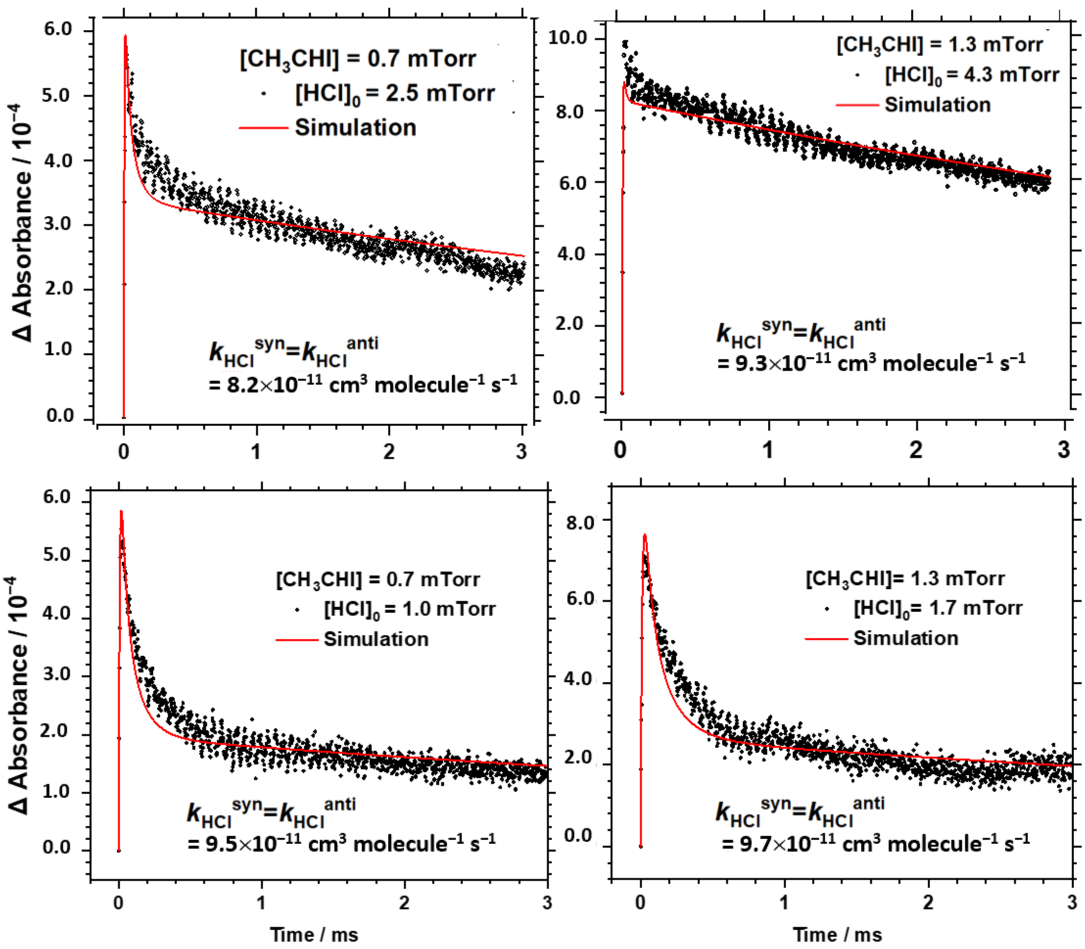

Figure S9. Comparison of temporal profiles probed near 1280 cm^{-1} with those of *syn*-CH_3CHOO + *anti*-CEHP fitted based on a kinetic model listed in Table 1 by assuming $k_{HCl}^{syn} = k_{HCl}^{anti}$. $[CH_3CHI_2]_0$, $[HCl]_0$ and fitted k_{HCl} are listed; P_T = 10 Torr and T = 298 K.

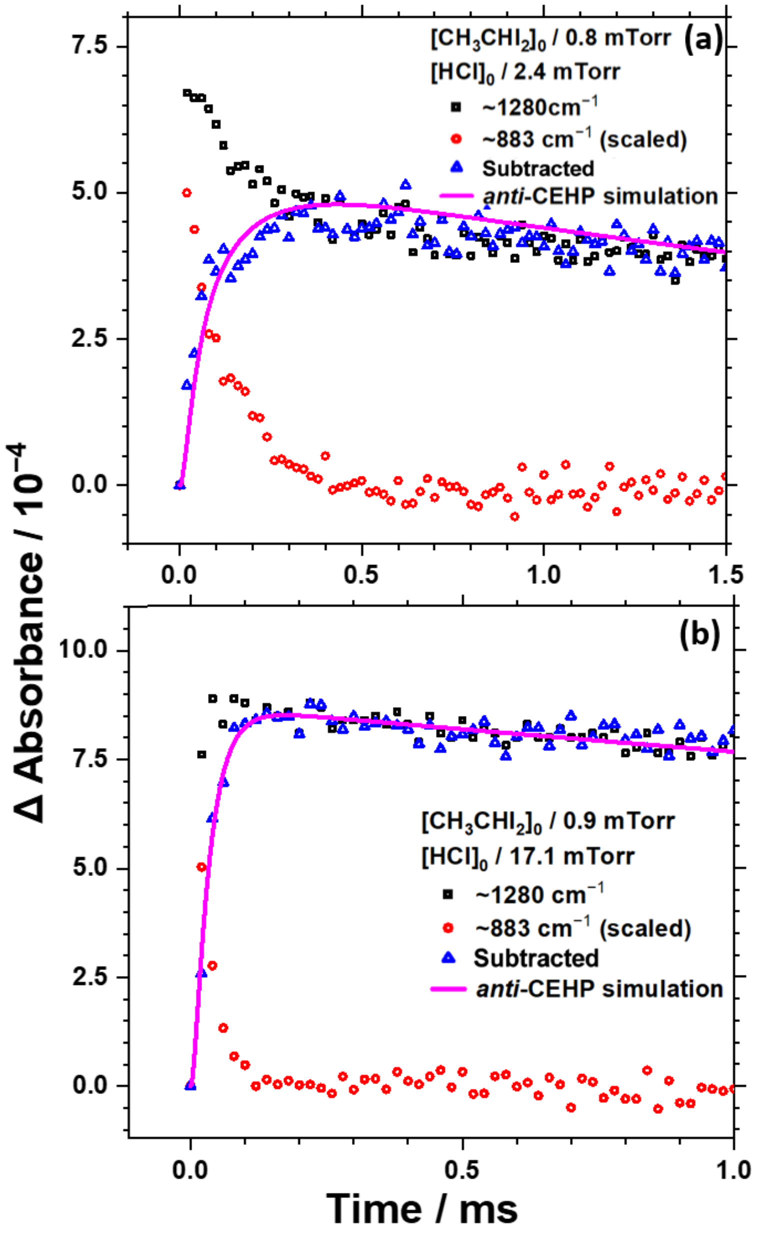

Figure S10. Derivation of temporal profiles of *anti*-CEHP and comparison with simulations. (a) $[HCl]_0$ = 8.0×10^{13} molecule cm^{-3}; (b) $[HCl]_0$ = 5.5×10^{14} molecule cm^{-3}. $[CH_3CHI_2]_0$ and $[HCl]_0$ are listed; P_T = 10 Torr and T = 298 K. Black squares (*syn*-CH_3CHOO + *anti*-CEHP) are measured near 1280 cm^{-1}; red circles are measured near 883 cm^{-1} and divided by $\sigma_{syn}^{883}/\sigma_{syn}^{1280}$ (= 6.8 ± 0.3) to represent absorbance of *syn*-CH_3CHOO near 1280 cm^{-1}. Blue triangles, representing temporal profiles of *anti*-CEHP, are the difference between the black and red symbols. The simulated profiles of *anti*-CEHP fitted according to Method B are shown in pink lines.

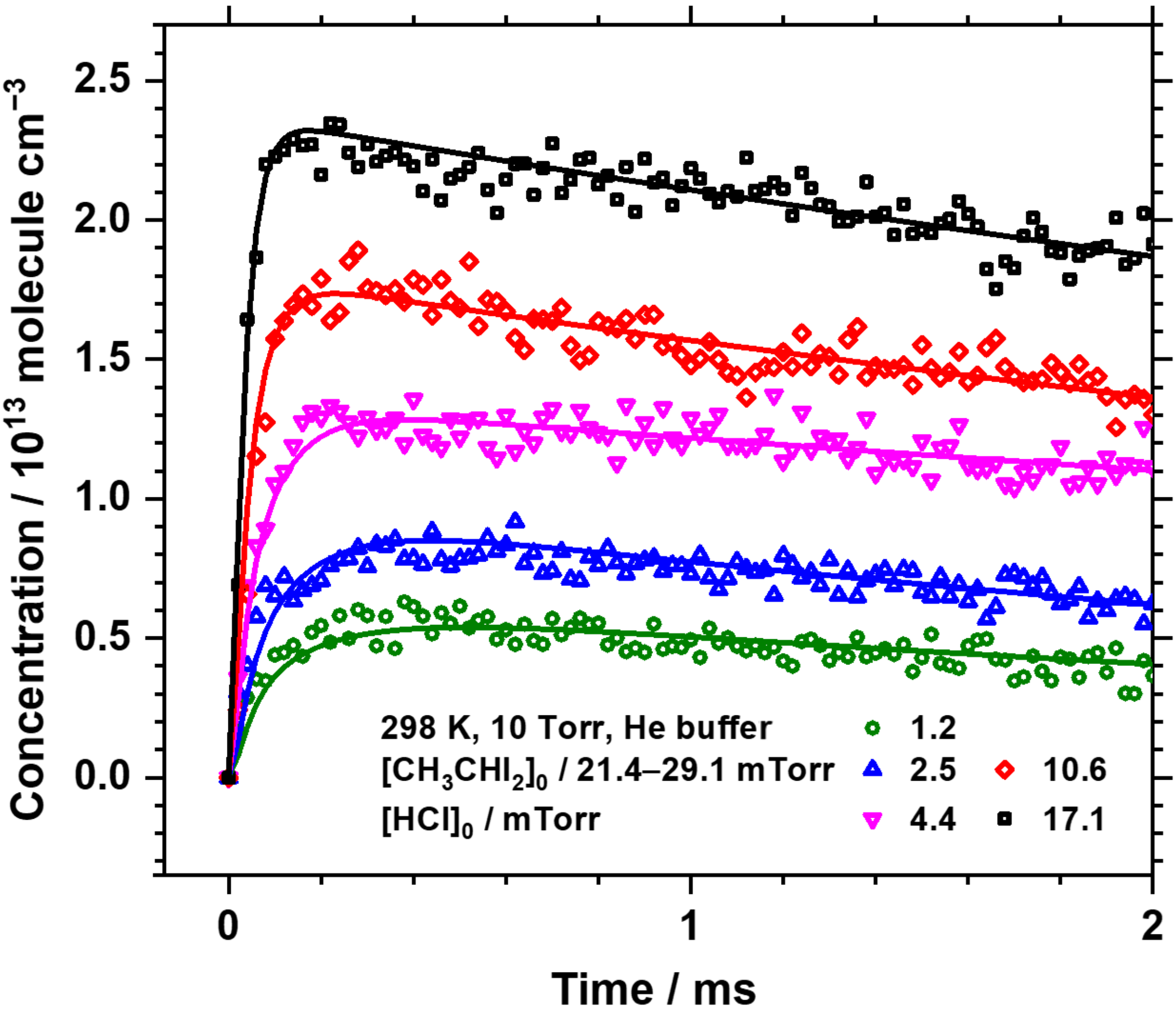

Figure S11. Comparison of derived temporal profiles of *anti*-CEHP in experimental set 2 with simulated profiles of *anti*-CEHP based on Method B. Total pressure P_T = 10 Torr and T = 298 K. Partial pressures of CH_3CHI_2 are (21.4–29.1) mTorr and those of HCl are (1.2–17.1) mTorr.

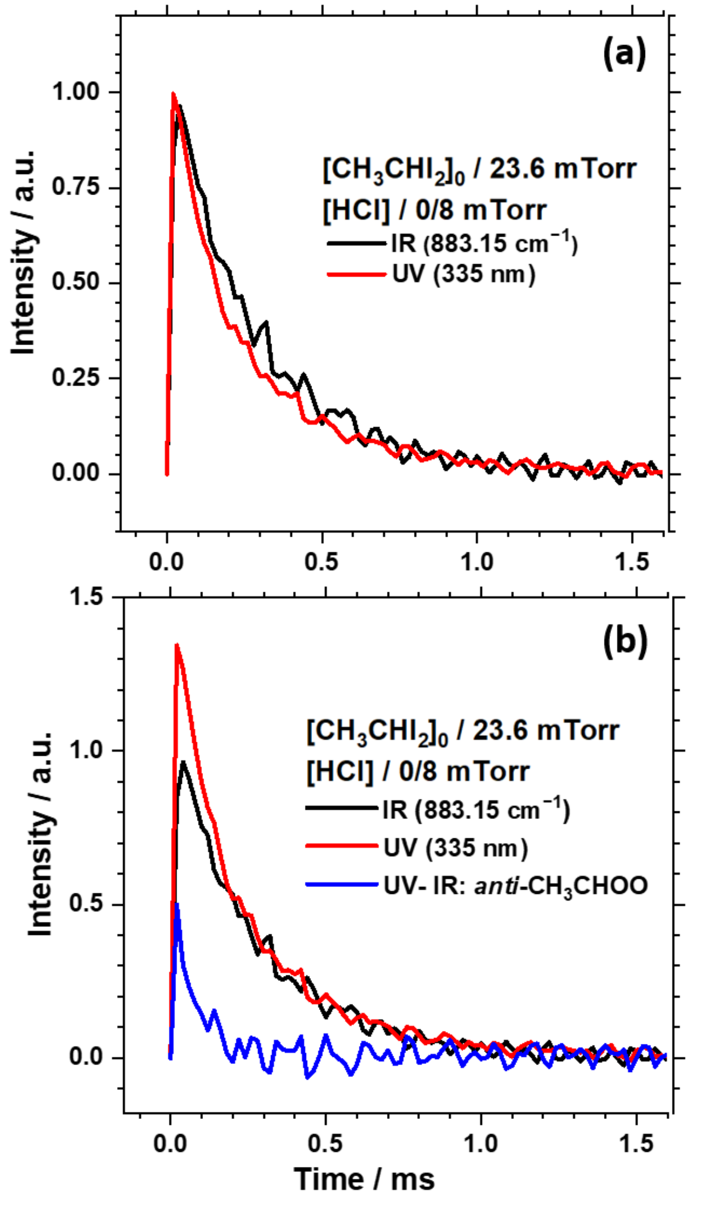

Figure S12. Derivation of temporal profiles of *syn*- and *anti*-CH_3CHOO from UV and IR probes in Method C. The IR (~883.15 cm^{-1}, black) and UV (335 nm, red) absorbance temporal profiles are compared in (a) with maxima normalized. (b) the slow-decay components of IR and UV profiles are matched. IR absorption is associated with *syn*-CH_3CHOO, whereas UV absorption associated with *syn*- and *anti*-CH_3CHOO. The *anti*-CH_3CHOO (blue) temporal profile was derived by subtracting the black curve from the red curve in (b). Total pressure P_T = 8 Torr and T = 298 K. Partial pressures of CH_3CHI_2 is 23.6 mTorr and that of HCl is 0.8 mTorr.

Supporting References

[1] Kao, T.-Y.; Chung, C.-A.; Lee, Y.-P. Rate Coefficient and Branching Ratio for the Formation of Criegee Intermediate *syn-/anti*-CH_3CHOO from $CH_3CHI + O_2$ and the Self-Reaction of *syn-/anti*-CH_3CHOO Determined with Simultaneous IR/UV Probes. *J. Phys. Chem. A* **2024**, *128*, 9453−9461.

[2] Howes, N. U. M.; Mir, Z. S.; Blitz, M. A.; Hardman, S.; Lewis, T. R.; Stone, D.; Seakins, P. W. Kinetic Studies of C_1 and C_2 Criegee Intermediates with SO_2 Using Laser Flash Photolysis Coupled with Photoionization Mass Spectrometry and Time Resolved UV Absorption Spectroscopy. *Phys. Chem. Chem. Phys.* **2018**, *20*, 22218−22227.

[3] Su, Z.-S.; Lee, Y.-P. Infrared Characterization of the Products of the Reaction between the Criegee Intermediate CH_3CHOO and HCl. *J. Phys. Chem. A* **2023**, *127*, 6902−6915.

[4] Western, C. M.; PGOPHER ver. 10.1, A Program for Simulating Rotational, Vibrational and Electronic Spectra, http://pgopher.chm.bris.ac.uk and https://doi.org/10.5523/bris.3mqfb4glgkr8a2rev7f73t300c. University of Bristol, Bristol (2018).

[5] Nakajima, M.; Endo, Y. Communication: Spectroscopic Characterization of an Alkyl Substituted Criegee Intermediate *syn*-CH_3CHOO through Pure Rotational Transitions. J. Chem. Phys. **2014**, *140*, 011101.

[6] Nakajima, M.; Yue, Q.; Endo, Y. Fourier-transform Microwave Spectroscopy of an Alkyl Substituted Criegee Intermediate *anti*-CH_3CHOO. *J. Mol. Spectrosc.* **2015**, *310*, 109−112.

[7] Lin, H.-Y.; Huang, Y.-H.; Wang, X.; Bowman, J. M.; Nishimura, Y.; Witek, H. A.; Lee, Y.-P. Infrared Identification of the Criegee Intermediates syn- and anti-CH3CHOO, and Their Distinct Conformation-Dependent Reactivity. Nat. Commun. **2015**, 6, No. 7012.

[8] Sheps, L.; Scully, A. M.; Au, K. UV Absorption Probing of the Conformer-Dependent Reactivity of a Criegee Intermediate CH_3CHOO. *Phys. Chem. Chem. Phys.* **2014**, *16*, 26701−26706.

[9] Ting, W.-L.; Chang, C.-H.; Lee, Y.-F.; Matsui, H.; Lee, Y.-P.; Lin, J. J.-M. Detailed Mechanism of the $CH_2I + O_2$ Reaction: Yield and Self-reaction of the Simplest Criegee Intermediate CH_2OO. *J. Chem. Phys.* **2014**, *141*, 104308.

[10] Gravestock, T. J.; Blitz, M. A.; Bloss, W. J.; Heard, D. E. A Multidimensional Study of the Reaction $CH_2I + O_2$: Products and Atmospheric Implications. *ChemPhysChem* **2010**, 11, 3928−3941.
